# Cep131 overexpression promotes centrosome amplification and colon cancer progression by regulating Plk4 stability

**DOI:** 10.1038/s41419-019-1778-8

**Published:** 2019-07-29

**Authors:** Dong Hyun Kim, Jong Seog Ahn, Ho Jin Han, Hye-Min Kim, Joonsung Hwang, Kyung Ho Lee, Hyunjoo Cha-Molstad, In-Ja Ryoo, Jae-Hyuk Jang, Sung-Kyun Ko, Jin Ok Yang, Hee Gu Lee, Sangku Lee, Eun Joo Song, Jin Young Kim, Yang Hoon Huh, Yong Tae Kwon, Nak-Kyun Soung, Bo Yeon Kim

**Affiliations:** 1 Anticancer Agent Research Center, Korea Research Institute of Bioscience and Biotechnology, Ochang, Cheongju 28116 Republic of Korea; 2 Department of Biomolecular Science, University of Science and Technology, Daejeon, 34113 Republic of Korea; 3 Korean Bioinformation Center, Korea Research Institute of Bioscience and Biotechnology, Daejeon, 34141 Republic of Korea; 4 Immunotherapy Convergence Research Center, Korea Research Institute of Bioscience and Biotechnology, Daejeon, 34141 Republic of Korea; 5 Molecular Recognition Research Center, Korea Institute of Science and Technology, Seoul, 02792 Republic of Korea; 6 Biomedical Omics Research, Korea Basic Science Institute, Ochang, Cheongju 28119 Republic of Korea; 7 Center for Electron Microscopy Research, Korea Basic Science Institute, Ochang, Cheongju 28119 Republic of Korea; 8 Protein Metabolism Medical Research Center, Department of Biomedical Sciences, College of Medicine, Seoul National University, Seoul, 03080 Republic of Korea

**Keywords:** Oncogenes, Cell division

## Abstract

The initiation of centrosome duplication is regulated by the Plk4/STIL/hsSAS-6 axis; however, the involvement of other centrosomal proteins in this process remains unclear. In this study, we demonstrate that Cep131 physically interacts with Plk4 following phosphorylation of residues S21 and T205. Localizing at the centriole, phosphorylated Cep131 has an increased capability to interact with STIL, leading to further activation and stabilization of Plk4 for initiating centrosome duplication. Moreover, we found that Cep131 overexpression resulted in centrosome amplification by excessive recruitment of STIL to the centriole and subsequent stabilization of Plk4, contributing to centrosome amplification. The xenograft mouse model also showed that both centrosome amplification and colon cancer growth were significantly increased by Cep131 overexpression. These findings demonstrate that Cep131 is a novel substrate of Plk4, and that phosphorylation or dysregulated Cep131 overexpression promotes Plk4 stabilization and therefore centrosome amplification, establishing a perspective in understanding a relationship between centrosome amplification and cancer development.

## Introduction

The centrosome, comprising a pair of centrioles and pericentriolar materials, is the microtubule-organizing center in animal cells. It is closely associated with microtubule-based cellular processes, such as cell division^1^. Since centrosomes ensure equal division of chromosomes into daughter cells, their number must be precisely controlled^2^. In fact, centrosome amplification, which results in extra centrosomes, frequently causes chromosome missegregation, aberration, and/or aneuploidy, which are common hallmarks of cancer^3,4^. Although whether centrosome amplification is directly related to cancer remains unclear, several studies have observed that it promotes early-onset tumorigenesis in conditions of p53 deficiency^5,6^ or in the presence of wild-type p53^7^, suggesting a close correlation between centrosome amplification and cancer development.

Centrosome duplication occurs only once every cell cycle and is tightly regulated by several key factors^8,9^. Among them, Plk4, also called Sak, is the first identified Polo-like kinase family member and an essential regulator of centrosome duplication^10,11^. At the beginning of centrosome duplication, Plk4 is recruited near the proximal end of the parental centriole^12^, and then directly interacts with and phosphorylates STIL (SAS5/Ana2) to facilitate formation of the STIL/HsSAS-6 complex^13–15^. This complex creates the crucial cartwheel structure that acts as the platform to elongate the new procentriole^16^. Thus, successful completion of the initial duplication step is determined by Plk4 kinase activity, promoting phospho-dependent interactions between STIL and HsSAS-6.

Previous studies have reported a self-inhibitory system for Plk4 through its formation of a homodimer that promotes auto-phosphorylation, thus creating a phospho-degron that is recognized by SCF/Slimb E3 ligase and undergoes ubiquitylation-dependent degradation^17–20^. Considering the importance of the Plk4 level and activity in centrosome maintenance^10^, this self-inhibitory system is necessary to tightly regulate Plk4 and prevent its uncontrolled accumulation or hyperactivation, which causes centrosome amplification. Interestingly, STIL not only binds Plk4 through the STIL-CC domain, but also activates and stabilizes Plk4 during the initiation of centrosome duplication^15,21,22^. Although Plk4, STIL, and HsSAS-6 are known to cooperatively create the cartwheel structure during initiation of centrosome duplication, the roles of other centrosomal proteins involved in regulating Plk4 activity and stability remain to be characterized.

In this study, we discovered a novel function of centrosomal protein 131 kDa (Cep131; also called AZI1) in centrosome duplication. Cep131 was originally identified as a centriolar satellite protein associated with genomic stability maintenance^23^ and cilia formation^24–26^. In addition, a recent study showed that elevated Cep131 levels induced by loss of deubiquitinase USP9X promoted centrosome amplification and breast cancer development^27^. However, how Cep131 levels influence centrosome amplification remains unclear. We demonstrate that Cep131 is a novel substrate of Plk4, and its phosphorylation facilitates interaction with STIL. Furthermore, Cep131 overexpression contributes to excessive recruitment of STIL to the centriole, which stabilizes Plk4, leading to centrosome amplification and cancer development.

## Results

### Cep131 overexpression leads to centrosome amplification associated with Plk4

**Fig. 1:**
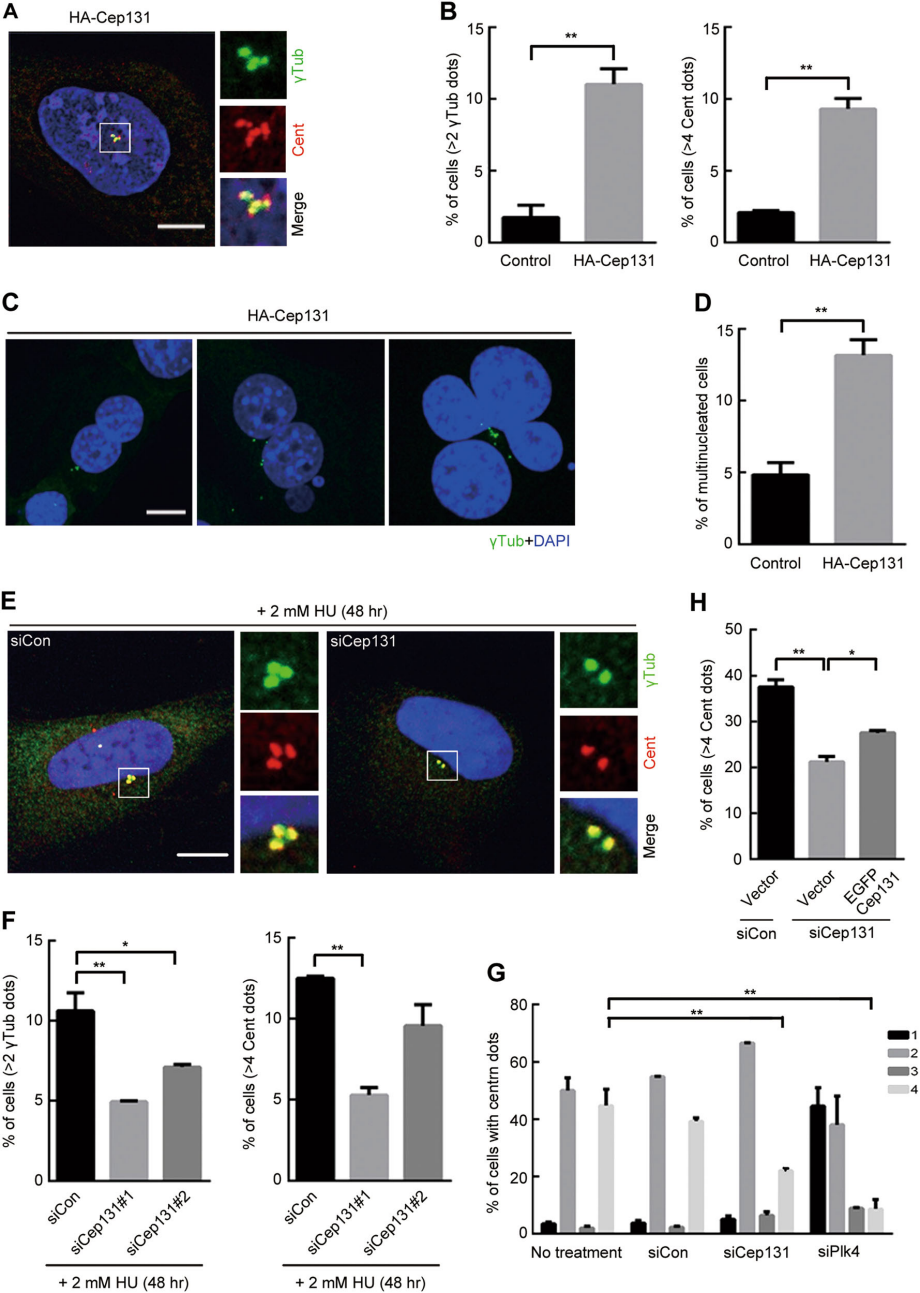
Cep131 overexpression induces centrosome amplification and genomic aberration. **a** Immunofluorescence analysis of centriole and centrosome amplification in U2OS cells stably expressing HA-Cep131. Cells were stained with anti-Cent (red) and anti-γTub (green) antibodies to visualize centrioles and centrosomes, respectively. Insets are approximately fivefold magnified at the centrosomal region. Scale bar, 10 μm. **b** Quantification of the proportion of cells with >2 centrosomes or >4 centrioles induced by stably expressing HA-Cep131. **c**, **d** Multinucleated cells were induced by HA-Cep131 overexpression in U2OS cells. DNA is visualized in blue. Scale bar, 10 μm. **e**, **f** U2OS cells were treated to 2 mM hydroxyurea (HU) for 48 h to induce centrosome amplification and were transfected with siCon or siCep131 duplexes. The graph shows quantification of cells with centrosome amplification (**f**). Insets are approximately fivefold magnified at the centrosomal region. Scale bar, 10 μm. **g** Quantification of the proportion of cells with centriole number after treatment of siCon, siCep131, and siPlk4. **h** Endogenous Cep131 was depleted by siRNA, and cells were transfected with EGFP vector or EGFP-Cep131, containing resistant sequences to siCep131. The graph shows quantification of four-centriole cells. Error bars in **b**, **d**, **f**, **g** represent means ± SEM from three independent experiments (*N* > 300 for each experiment). *P < 0.05 and **P < 0.01, unpaired Student’s *t* test

To clarify the effect of Cep131 protein levels on centrosome duplication^27^, we prepared human U2OS cells stably overexpressing HA-tagged Cep131 (HA-Cep131) (Fig. S1a). Elevated Cep131 enhanced centriole and centrosome amplification by ~10%, as indicated by the centriole marker Centrin (Cent) and centrosome marker γ-tubulin (γTub), respectively (Fig. 1a, b and S1a). In contrast, centriole and centrosome amplification were blocked by siRNA-induced Plk4 knockdown (Fig. S1b, c). In addition, HA-Cep131 overexpression increased the number of multinucleated cells (Fig. 1c, d), suggesting that Cep131 promotes centrosome amplification and genomic aberration. Next, we assessed the effect of Cep131 on centrosome amplification induced by S-phase arrest or Plk4 overexpression. Long-term treatment of hydroxyurea (HU) (Fig. 1e, f) and conditional Plk4 overexpression (Fig. S1e, f) increased centriole and centrosome amplification, whereas loss of endogenous Cep131 decreased centrosome amplification (Fig. S1d, f).

To examine Cep131’s role in centrosome formation, we measured the populations of cells with different numbers of centrioles after Cep131 knockdown. The proportion of four-centriole cells decreased moderately in the siCep131 group compared with that in the untreated and siCon groups (Fig. 1g), indicating the hindering effect of Cep131 knockdown on centrosome duplication. Re-expression of GFP-tagged Cep131 (GFP-Cep131) containing an siRNA-resistant sequence partially rescued the proportion of four-centriole cells (Fig. 1h). These data suggest that Cep131 is associated with Plk4-dependent centrosome duplication; thus, its overexpression causes centrosome amplification and genomic aberration.

### Cep131 asymmetrically localizes at the centriole

Cep131 was originally identified as a centriolar satellite protein clustering near the centrosomes^23,28–30^. Our immunofluorescence analysis suggested that endogenous Cep131 was located near the centrosome throughout the cell cycle (Fig. S2a). We had noted that the Cep131 signal also presented the core of centrosome (centriole) (Fig. S2a), and therefore investigated the localization of Cep131 at the centriole. To this end, U2OS cells were subjected to signal extraction^31^ to reduce the cytoplasmic background of γTub. After signal extraction, immunofluorescence analysis showed that the Cep131 signal weakened near the centrosome, while it was brighter at the centriole, which co-stained with Cent (Fig. S2b). This signal of endogenous Cep131 at the centriole was very weak or undetectable during mitosis (Fig. S2b), indicating cell cycle-dependent Cep131 localization at the centriole. Consistence with these results, centriolar Cep131 localization was also confirmed by ectopic expression of GFP-Cep131 co-stained with Cent or Plk4 (Fig. S2c).

**Fig. 2:**
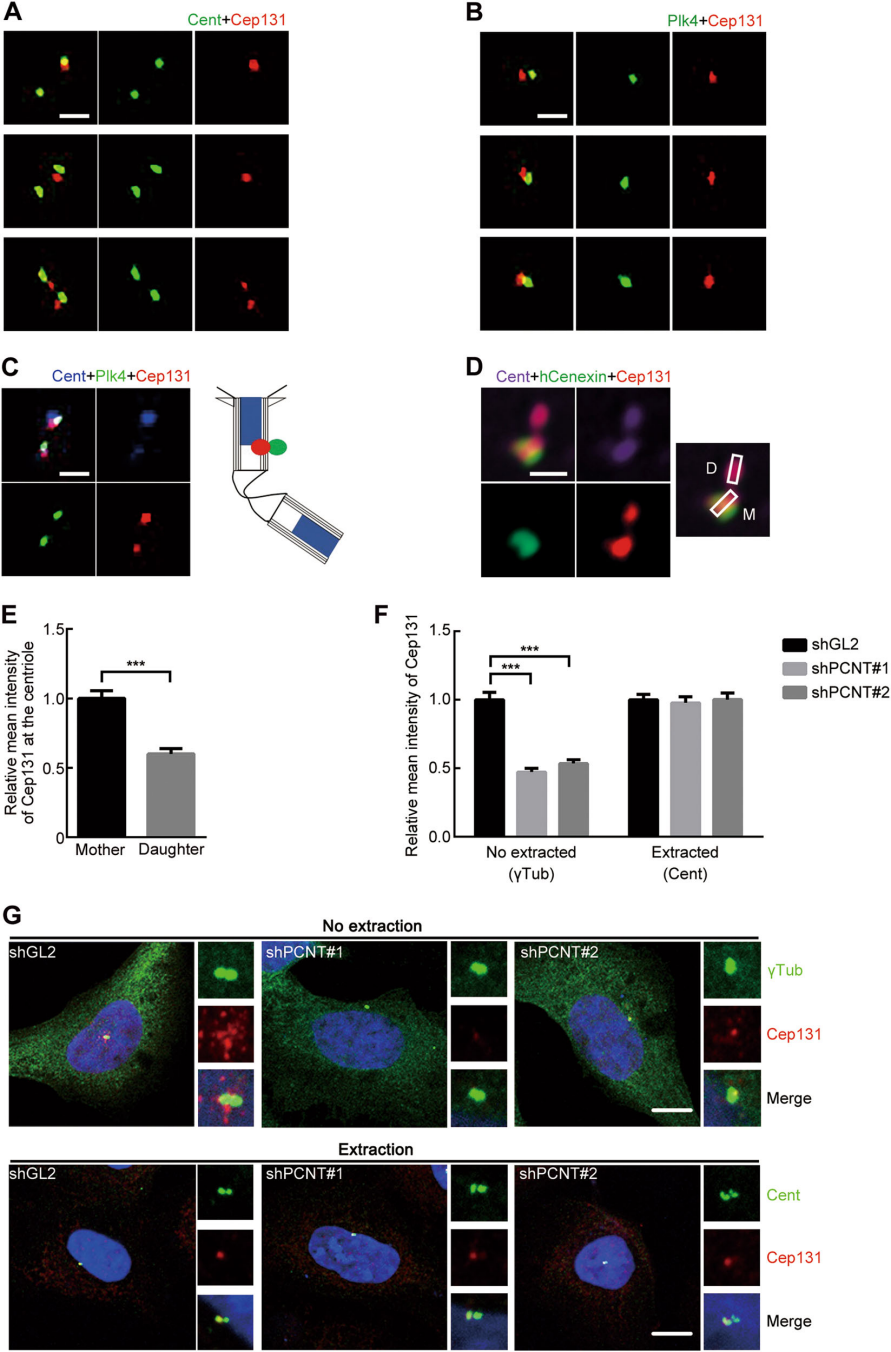
Localization of Cep131 at the centriole. Immunofluorescence analysis of direct localization of Cep131 at the centriole. U2OS cells were co-stained for Cep131 (red) with centriolar marker proteins, such as anti-Cent **a** and Plk4 **b**. Scale bar, 0.5μm. **c** Triple staining of U2OS cells with antibodies against anti-Cent (blue), Plk4 (green), and Cep131 (red). Schematic illustration of the localization of Cep131 at the outer wall of centriole (right panel). Scale bar, 0.5 μm. **d** Asymmetrical localization of Cep131 at the mother centriole. Cep131 co-stained with Cent (far-red) and mother centriole-enriched protein, hCenexin (green). Schematic illustration to recognize each centriole, mother (M) and daughter (D) centriole. Scale bar, 0.5 μm. **e** Quantification of fluorescence intensity of Cep131 at each centriole (mother and daughter). Around 50 centrioles from three independent experiments were measured for each condition. ***P < 0.001, unpaired Student’s *t* test. **f**, **g** U2OS cells were treated with shGL2 (control) or shPCNT duplexes and were fixed by two different methods, common fixation (No extraction) and signal extraction (Extraction), to verify the localization of Cep131 at centriolar satellites and the centriole, respectively. Around 50 cells from three independent experiments were measured for each condition. ***P < 0.001, unpaired Student’s *t* test

To define the specific localization of Cep131 at the centriole, we applied cells with super-resolution radial fluctuations (SRRF), which allow super-resolution imaging approaching single-molecule localization analysis^32^. SRRF imaging revealed that the Cep131 and Cent showed the two proteins close to one another (Fig. 2a). Moreover, the generated antibody against Plk4 (Fig. S2d) also closely co-localized with Cep131 at the centriole (Fig. 2b). As previously reported^12,33^, most cells exhibited centriolar Plk4 localized in a dot-like pattern on the outer wall of the centriole, while the Cep131 signal exhibited a similar pattern, only partially overlapping with Plk4 (Fig. 2c).

We also observed that the Cep131 signal was much brighter on one of two centrioles (Fig. 2a, c). To clarify this, we assessed the localization of Cep131 relative to hCenenxin^34^ and Ninein^35^, which are dominantly enriched at mother centriole appendages. The bright Cep131 signal was mainly observed at the mother centriole, as revealed by co-localization with hCenexin (Fig. 2d), as well as with three Ninein dots (Fig. S3a). In contrast, most cells presented a weak level of Cep131 signal at the daughter centriole (Fig. 2d, e).

We then investigated whether knockdown of pericentrin (PCNT), a major factor in Cep131 recruitment to centriolar satellites^23^, affected centriolar Cep131 localization. In U2OS cells treated with shPCNT duplex, the Cep131 signal at the centrosome and in its vicinity was nearly eliminated (Fig. 2f, g). However, PCNT depletion did not change centriolar Cep131 localization (Fig. 2f, g), indicating that centriolar loading of Cep131 is not associated with PCNT. Furthermore, loss of several centriolar proteins, including Cep152, Cep192, ATF5, hCenexin, Cep250, rootletin, Plk4, and STIL, did not affect centriolar Cep131 localization (Fig. S3b, c). Unlike Cep131 recruitment at centriolar satellite, unidentified protein in centrosome network may be involved in Cep131 loading at the centriole. Taken together, these observations indicate that Cep131 directly and asymmetrically localizes at the centriole independently of PCNT.

### Plk4 directly interacts with Cep131

Since Cep131 plays an essential role in centrosome duplication (Fig. 1), we assessed the functional enrichment of Cep131 and Plk4 in a gene interaction network using Ingenuity Pathway Analysis (IPA; Ingenuity Systems). The informatics database showed that Cep131 and Plk4 jointly contributed to shared functions, which identified from experimentally determined information^36^, curated databases, and text-mining data^37,38^.

**Fig. 3:**
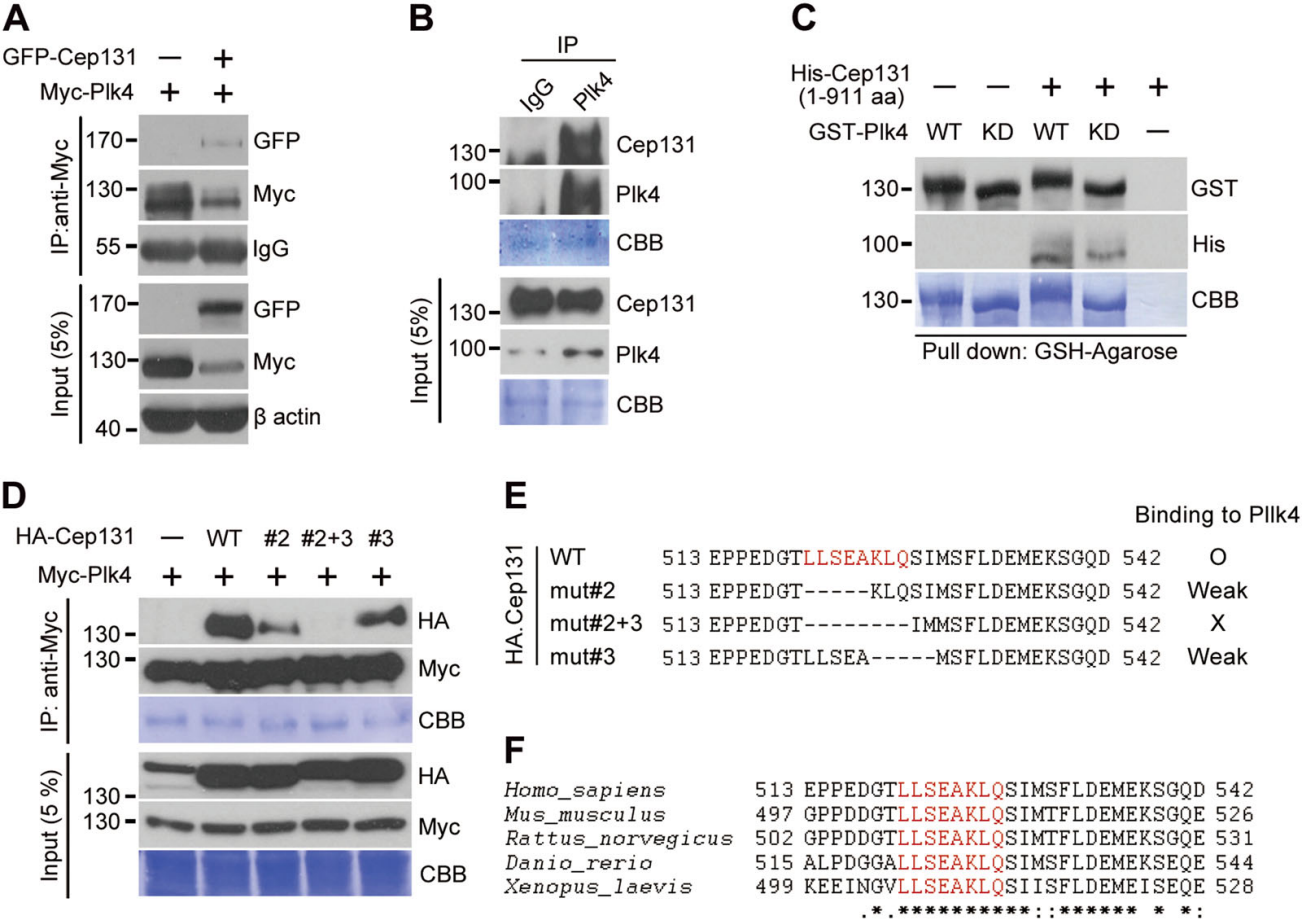
Plk4 directly interacts with Cep131. **a** HEK293T cells co-expressing Myc-Plk4 and GFP-Cep131 were immunoprecipitated using Myc-magnetic beads, and the protein levels were observed by immunoblotting. **b** Endogenous Plk4 extracted from U2OS cells was immunoprecipitated by anti-Plk4, and total and pulled down proteins were observed by immunoblotting. **c** Bacterially generated GST-Plk4-WT or KD were incubated with His-Cep131 (1-911 aa) and pulled down using GSH-agarose. **d**, **e** HEK293T cells co-expressing Myc-Plk4 and HA-Cep131 deletions, including #mut2, #mut2+3, and #mut3, as illustrated in **e**, were immunoprecipitated, and protein levels were observed. **f** Alignment of amino acids from 513–542 in human Cep131 corresponded to several other species. Plk4-binding motif (PBM) is highlighted in red. Asterisk, fully conserved residue; colon, conserved residue; period, semi-conserved residue. A Coomassie (CBB) staining use as a loading control in **b**–**d**

We then examined the physical association between Cep131 and Plk4 in HEK293T cells expressing Myc-Plk4 and GFP-Cep131. Co-immunoprecipitation analysis demonstrated that Plk4 interacted with Cep131 (Fig. 3a, b). Moreover, bacterially expressed Plk4 (GST-Plk4) favorably pulled down His-tagged Cep131 (Fig. 3c). A kinase-dead mutant of Plk4 (KD, K41R) interacted similarly with Cep131 (Fig. 3c), indicating that Plk4 interacts with Cep131 regardless of kinase activity.

To determine which region of Cep131 is required for Plk4 binding, we generated HA or GFP-tagged Cep131 fragments and performed co-immunoprecipitation with Myc-Plk4 (Fig. S4a–d). We finally identified residues 520–527 (LLSEAKLQ) as the Cep131 Plk4-binding motif (PBM), since this sequence was essential to the interaction with Plk4 (Fig. 3d, e and S4e, f). This PBM is highly conserved from *Xenopus laevis* to *Homo sapiens* (Fig. 3f). Our results demonstrate that Cep131 directly binds Plk4, and that this interaction requires the specific residues in the PBM.

### Cep131 is a novel substrate of Plk4

**Fig. 4:**
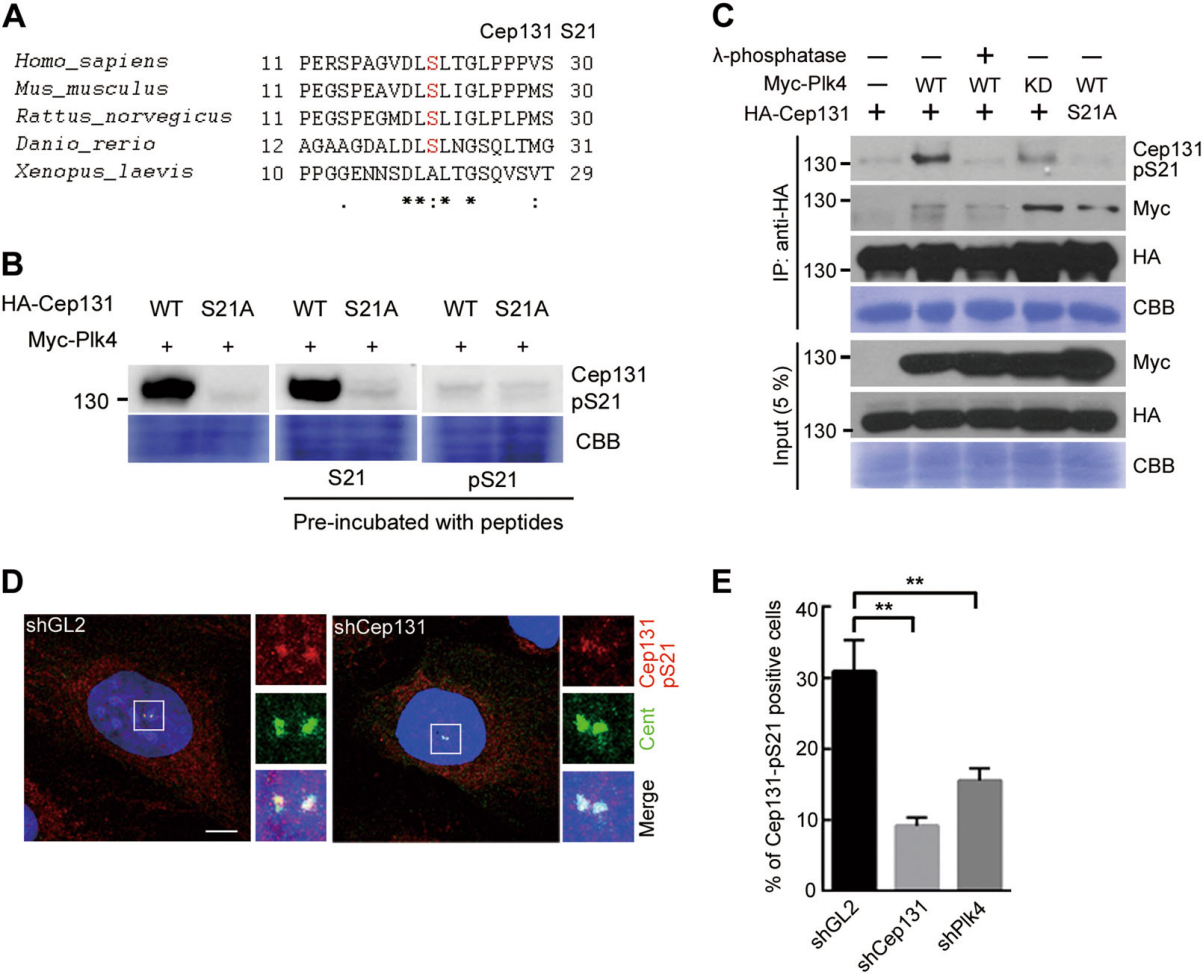
Cep131 is phosphorylated by Plk4. **a** Alignment of amino acids in human Cep131 and several other species. The phosphorylation sites at residues S21 by Plk4 are highlighted in red. Asterisk, fully conserved residue; colon, conserved residue; period, semi-conserved residue. **b** Peptide competition assay to verify the specificity of anti-Cep131-pS21 antibody. Lysates from HEK293T cells expressing Myc-Plk4 with HA-Cep131-WT or S21A were incubated with non- or phosphopeptides targeting Cep131-S21. After incubation, phosphorylation status was analyzed by immunoblotting using Cep131-pS21 antibody. **c** HEK293T cells co-expressing HA-Cep131-WT with Myc-Plk4-WT or KD were immunoprecipitated using HA-magnetic beads, followed by incubation with λ-phosphatase at 30 °C for 30 min. Phosphorylation status was then observed by immunoblotting using anti-Cep131-pS21 antibody. **d** Immunofluorescence analysis of co-localization of phosphorylated Cep131 at the centriole. U2OS cells were treated with shGL2 (control) or shCep131 and were co-stained for Cep131-pS21 (red) and Cent (green). **e** Quantification of Cep131-pS21-positive cells. Error bars represent means ± SEM from three independent experiments (*N* > 300 for each experiment). **P < 0.01, unpaired Student’s *t* test. CBB staining use as a loading control in **b**, **c**

To understand the functional association between Cep131 and Plk4, we examined whether Plk4 directly phosphorylates Cep131. Using an in vitro kinase assay and mass spectrometry, six serine/threonine candidate sites were discovered (Fig. S5a, b). Finally, residues S21 and T205 at the N terminus were confirmed as Plk4-specific phosphorylation sites, since their mutation to non-phosphorylatable alanine (S21A and T205A) reduced Plk4-dependent phosphorylation (Fig. S5c). Notably, S21 in Cep131 is conserved among vertebrates from *Danio rerio* to *H. sapiens* (Fig. 4a), while T205 is not (Fig. S5d).

To further verify Cep131 phosphorylation in cells, we generated antibodies against a Cep131 phosphopeptide comprising S21 (Cep131-pS21) (Fig. 4b). After co-expressing HA-Cep131 and Myc-Plk4 in HEK293T cells, phosphorylated Cep131 was detected, but clearly abolished upon treatment with λ-phosphatase and co-expression of Myc-Plk4-KD or HA-Cep131-S21A (Fig. 4c). Interestingly, deletion of the PBM in Cep131 significantly decreased its phosphorylation (Fig. S5e), suggesting that Cep131 phosphorylation by Plk4 is required for the interaction of both proteins through the specific PBM. Furthermore, immunostaining revealed that phosphorylated Cep131 was localized at the centriole (Fig. 4d), and completely disappeared after depleting endogenous Cep131 or Plk4 (Fig. 4e). These results suggest that Plk4 directly phosphorylates Cep131 on residues S21 and T205, with phosphorylation occurring at the centriole.

### Plk4 kinase activity promotes the interaction of Cep131 with STIL to recruit STIL to the centriole

**Fig. 5:**
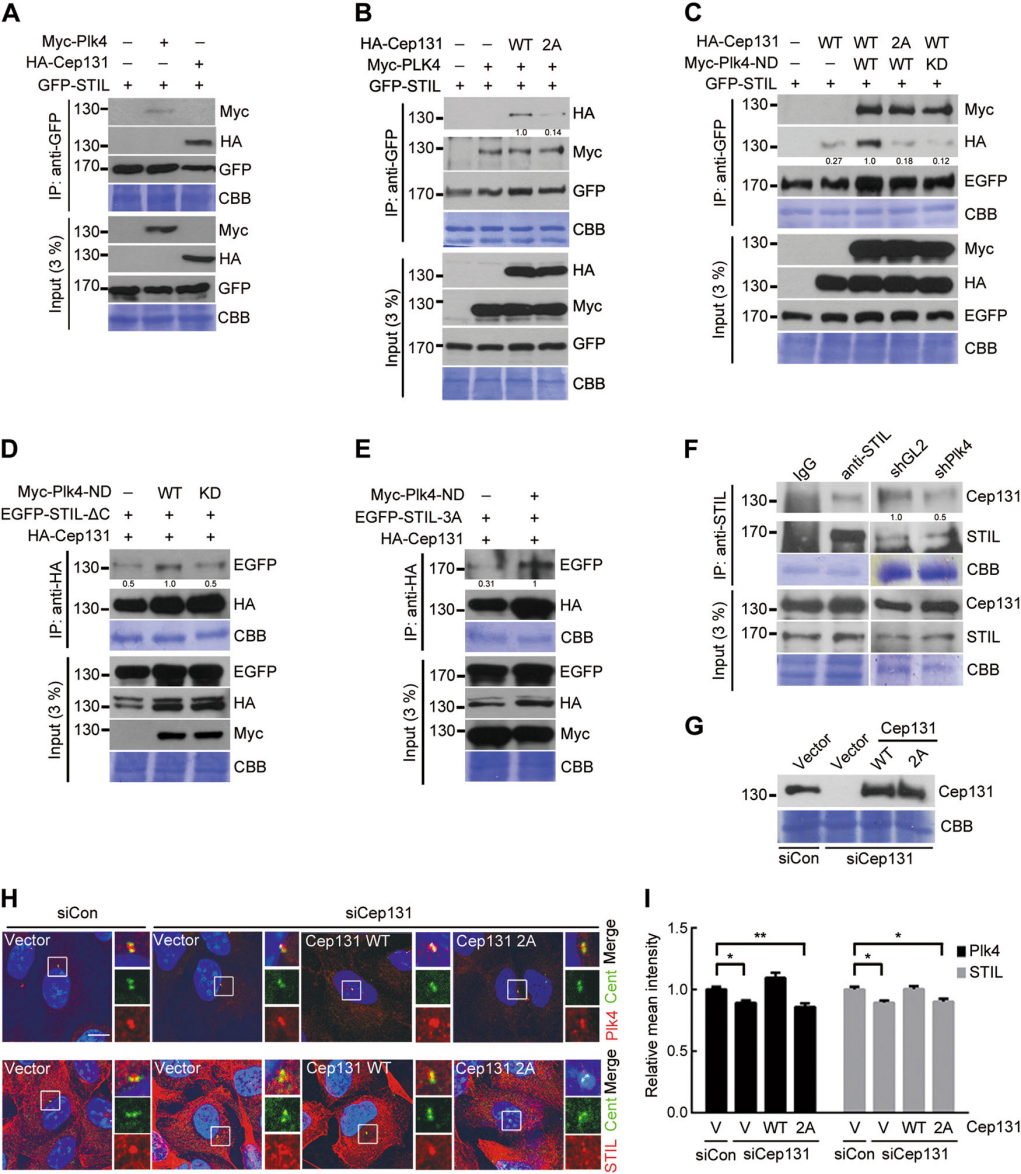
Phosphorylation of Cep131 by the Plk4 promotes interaction with STIL. Immunoblotting analysis of immunoprecipitated samples from HEK293T cells co-expressing GFP-STIL with several constructs, such as Myc-Plk4 or GFP-Cep131 **a**, Myc-Plk4 with GFP-Cep131 (WT or 2A) **b**, and Myc-Plk4-ND (nondegradable; constitutive active form) with GFP-Cep131 (WT or 2A) **c**. After immunoprecipitation using GFP-magnetic beads, protein levels were detected using the indicated antibodies. HEK293T cells co-expressing HA-Cep131-WT and GFP-STIL-ΔC (truncated C terminus of STIL, 1-831 aa) (**d**) or GFP-STIL-3A (alanine mutants on residue S1015, S1062, and S1070) (**e**) were immunoprecipitated using HA-magnetic beads, and the proteins levels were observed by immunoblotting. **f** Endogenous STIL extracted from U2OS cells was immunoprecipitated, and total and pulled down proteins were observed by immunoblotting. Quantification of band intensity was measured by ImageJ (NIH, Bethesda, MD, USA) and indicated as ratios in **b**–**f**. **g** Immunoblotting analysis shows protein levels of Cep131. U2OS cells with stable expression of untagged Cep131-WT and 2A containing resistant sequences to siRNA were transfected with siCon or siCep131 to eliminate endogenous Cep131. **h** Immunofluorescence analysis Plk4 variations (top panel) and STIL (bottom panel) levels at the centriole in U2OS cells transfected as in **g**. Insets are approximately fivefold magnified at the centrosomal region. Scale bar, 10 μm. **i** The graph shows quantification of the fluorescence intensity of Plk4 (left) and STIL (right) at the centriole. More than 50 centrioles were measured for each condition. *P < 0.05 and **P < 0.01, unpaired Student’s *t* test. CBB staining use as a loading control in **a**–**g**

Previous studies reported that Plk4 kinase activity plays a crucial role not only in forming the STIL/SAS-6 complex, but also in recruiting STIL to the centriole^15,21^. We therefore hypothesized that Cep131 phosphorylation by Plk4 might affect centriolar recruitment of STIL. To verify this, we generated and expressed HA-Cep131 with a double-mutation on residues S21 and T205 to alanine (HA-Cep131-2A), eliminating the effect of Plk4-induced phosphorylation. Immunoprecipitation analysis revealed that Cep131 directly bound not only Plk4 but also STIL (Fig. 5a). Interestingly, the phosphorylation mutant HA-Cep131-2A (double mutation on residues S21 and T205 to alanine) showed diminished binding to STIL compared with that of HA-Cep131-WT (Fig. 5b). We next assessed the effect of Plk4 kinase activity on the Cep131/STIL interaction. Cells expressing constitutively active Plk4 (nondegradable, ND)^20^ exhibited strikingly elevated Cep131/STIL interaction, which was abolished upon expression of HA-Cep131-2A or Myc-Plk4-KD (Fig. 5c). Moreover, phosphomimetic peptides, including S21D and E, increased Cep131 interaction with STIL, while T205E, but not the D mutant, somewhat influenced it (Fig. S6a). These results suggest that S21 is the major site responsible for the Cep131/STIL interaction, although Cep131-T205 has some marginal effect.

Given the observations that several residues on STIL constituting the STAN motif can be directly phosphorylated by Plk4^14,15^, we next examined the association between the STIL-STAN motif and Cep131/STIL interaction. To this end, we used two versions of STIL: STAN motif-deleted STIL (ΔC) and alanine mutants of the Plk4-induced phosphorylation sites (3A). Co-immunoprecipitation analysis demonstrated that both ΔC and 3A versions of STIL strongly interacted with Cep131 when Myc-Plk4-ND was co-expressed (Fig. 5d, e), indicating that STIL phosphorylation is not involved in the Cep131/STIL interaction. Furthermore, endogenous STIL extracted from U2OS cells directly interacted with Cep131, which markedly decreased upon treatment with shPlk4 (Fig. 5f).

We then investigated whether phosphorylated Cep131 affected STIL recruitment to the centriole. To measure the centriolar localization of endogenous STIL, U2OS cells stably expressing untagged exogenous Cep131-WT or 2A containing a resistant sequence against siRNA were treated with siCep131 to eliminate endogenous Cep131 (Fig. 5g and S6b). Depletion of Cep131 using siRNA led to a slight loss of STIL at the centrioles (Fig. 5h), and re-expression of Cep131-WT rescued the signal intensity of STIL at the centrioles (Fig. 5i). In contrast, Cep131-2A expression did not affect STIL recruitment (Fig. 5i). Taken together, these results suggest that Plk4 kinase activity induces Cep131 phosphorylation followed by interaction with STIL, consequently leading to the recruitment of STIL to the centrioles.

### Cep131 overexpression increases Plk4 stability through excessive recruitment of STIL to centrioles

Since centriolar STIL recruitment during initiation of centrosome duplication stabilizes Plk4 by preventing proteolytic degradation^14,15^, we assessed whether Cep131 stabilizes Plk4 through centriolar recruitment of STIL. As shown in Fig. 5h, i, immunofluorescence imaging revealed that the signal intensity of Plk4 at the centriole changed depending on the abundance of STIL, which was modulated by manipulating Cep131 levels.

**Fig. 6:**
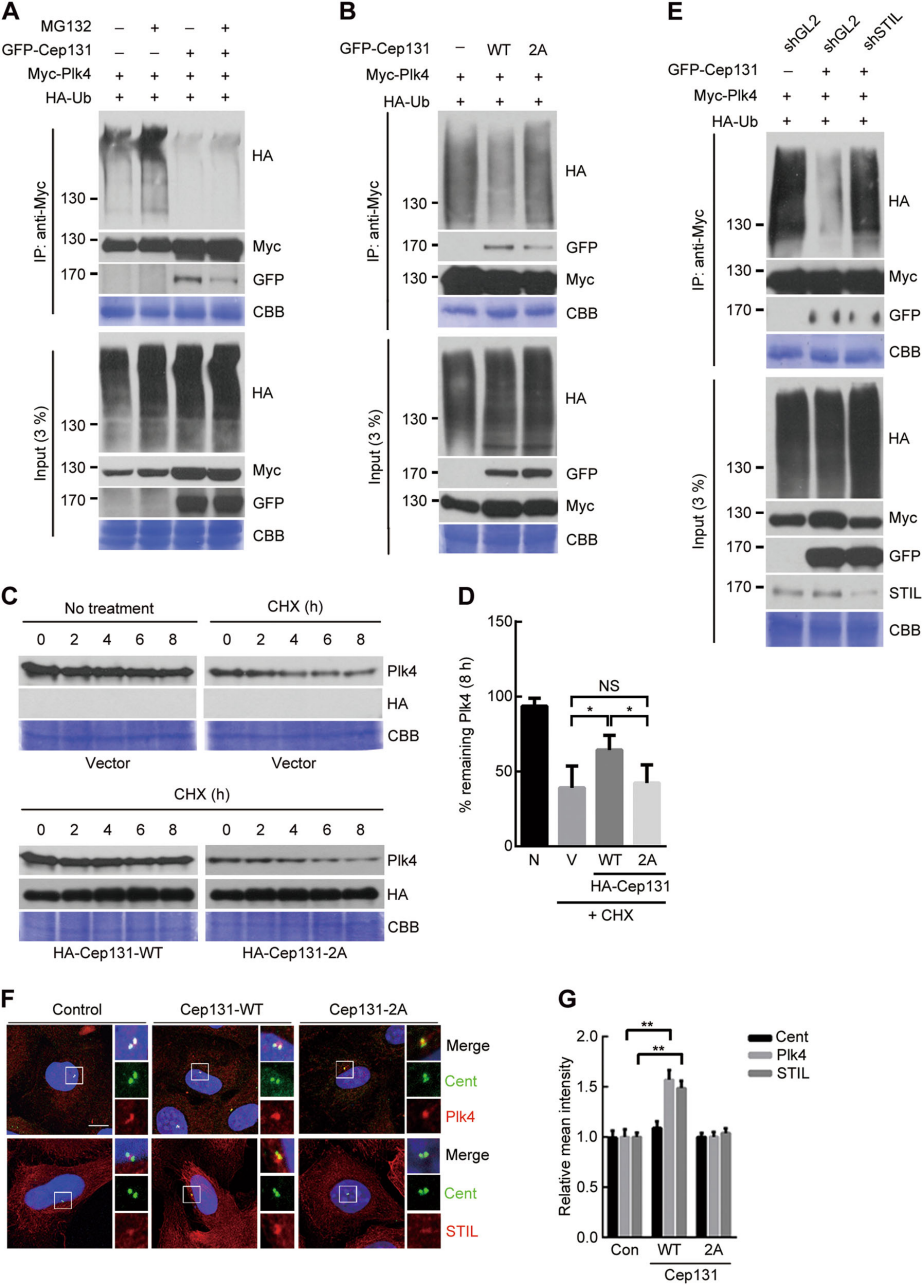
Cep131 overexpression induces Plk4 stabilization and STIL accumulation at the centriole. **a**, **b** HEK293T cells co-expressing Myc-Plk4 and HA-Ub and/or GFP-Cep131 were treated with MG132 for 6 h and immunoprecipitated. Ubiquitylation properties were analyzed by immunoblotting. **c** U2OS cells with doxycycline-inducible expression of Myc-Plk4 were treated with 10 μM cycloheximide (CHX) for 8 h. Every 2 h, cells were harvested and analyzed by immunoblotting. **d** Quantification of Plk4 band intensity measured with ImageJ. Error bars represent means ± SD from three independent experiments. **P < 0.01, unpaired Student’s *t* test. NS, not significant. **e** HEK293T cells stably expressing shGL2 (control) or shSTIL were transfected with HA-Cep131-WT or 2A and immunoprecipitated. Ubiquitylation properties were analyzed by immunoblotting. **f** Immunofluorescence analysis of excessive accumulations of Plk4 (top panel) and STIL (bottom panel) at the centriole in U2OS cells transfected with Cep131-WT or 2A. Insets are approximately fivefold magnified at the centrosomal region. Scale bar, 10 μm. **g** Quantification of fluorescence intensity of Cent (left), Plk4 (middle), and STIL (right) at the centriole. Over 50 centrioles were measured for each condition. **P < 0.01, unpaired Student’s *t* test. CBB staining use as a loading control in **a**, **b**, **c**, **e**

For biological evidence, we examined ubiquitylation properties of Plk4 under Cep131-expressing conditions. Immunoprecipitation analysis showed that GFP-Cep131 overexpression significantly reduced ubiquitylated Plk4 regardless of the presence of MG132, a proteasome inhibitor (Fig. 6a), whereas, in striking contrast, GFP-Cep131-2A did not affect ubiquitylation (Fig. 6b and S7a, b). Moreover, STIL overexpression reduced ubiquitylated Plk4 (Fig. S7a), suggesting that this as well as Cep131 protect Plk4 from degradation. To confirm the protective role of Cep131, we assessed the Plk4 degradation rate in U2OS cells under cycloheximide (CHX) treatment. Conditional expression of Myc-Plk4 (Fig. S1e) showed a gradual degradation of Plk4 (Fig. 6c, d). However, Plk4 degradation was delayed by HA-Cep131-WT overexpression, whereas HA-Cep131-2A had marginal effect (Fig. 6c, d). Thus, Cep131 inhibits Plk4 ubiquitylation and prevents its degradation.

Given that Cep131 was responsible for recruiting STIL to centrioles through Plk4-dependent phosphorylation (Fig. 5), we investigated whether the inhibitory effect of Plk4 degradation induced by Cep131 overexpression depended on the presence of STIL at the centrioles. To this end, endogenous STIL was silenced in HEK293T cells using shSTIL for the assessment of ubiquitylated Plk4. Although Cep131 overexpression markedly impaired Plk4 ubiquitylation, this impairment was not achieved without STIL (Fig. 6e), indicating that Cep131 levels regulate Plk4 stability through recruitment of STIL to the centriole.

Next, we examined the biological relevance of controlling Plk4 stability. Immunostaining data revealed that Cep131-WT but not Cep131-2A overexpression significantly increased (~1.5-fold) the signal intensity of both STIL and Plk4 at the centriole (Fig. 6f, g). In addition, following an increase of Plk4 stability by Cep131 overexpression, centrosome amplification was elevated over 10%, whereas Cep131-2A overexpression had no detectable effect (Fig. S7c, d). These results demonstrate that Cep131 overexpression results in excessive recruitment of STIL, leading to Plk4 stabilization and centrosome amplification.

### Cep131 overexpression promotes colon cancer progression

We explored the differential gene expression pattern of Cep131 in normal and cancer samples. Based on data from the Human Protein Atlas (https://www.proteinatlas.org), Cep131 expression has been confirmed in several organs, including the placenta, cerebellum, stomach, testis, and colon. Notably, Cep131 is within the 1% of genes most upregulated in bladder and colorectal cancer studies, as analyzed by the Oncomine database (http://www.oncomine.org) (Table S1 and Fig. S8a). Moreover, analysis of differential gene expression using The Cancer Genome Atlas (TCGA) RNASeq data set revealed that overall Cep131 expression level based on average normalized read count is high in several cancers, such as uterine, bladder, lung, kidney, breast, and colon (Table S2 and Fig. S8b). Both database analyses demonstrated significantly increased expression of Cep131 in bladder and colon cancers.

**Fig. 7:**
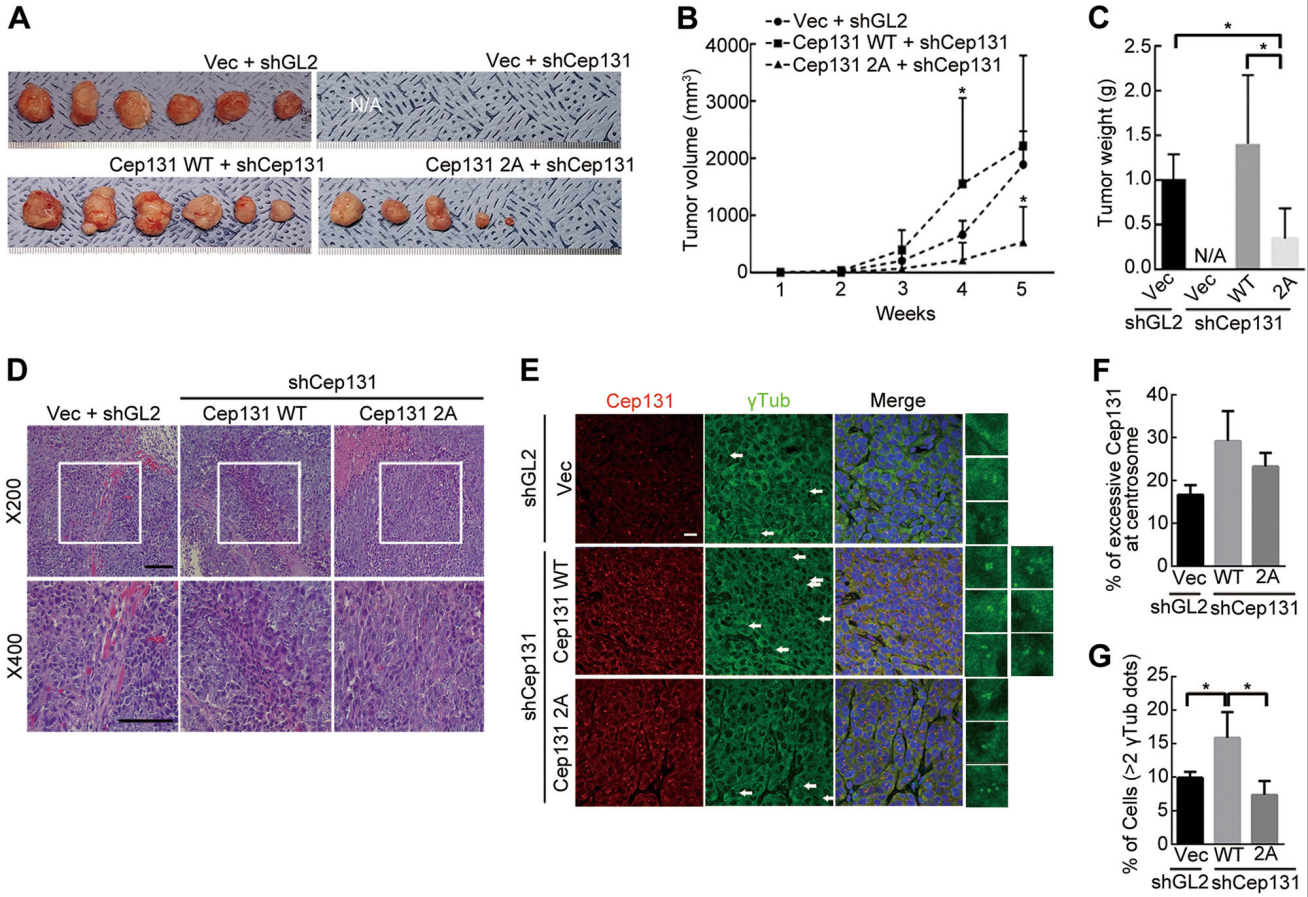
Overexpression of Cep131 is closely associated with colon cancer progression. **a** HCT116 cells stably expressing vector or untagged Cep131-WT or 2A were infected with shGL2 (control) or shCep131 to eliminate endogenous Cep131 and transplanted into nude mice. After 5 weeks, tumors had grown to maximum sizes and were removed. N/A, not available. Quantification of transplanted tumor volumes (**b**), measured weekly, and weight (**c**). Error bars represent means ± SD (*N* = 6 for each group). *P < 0.05, unpaired Student’s *t* test. **d** Tumors were stained with hematoxylin (violet) and eosin (pink) to indicate the nucleus and cytoplasm, respectively. Scale bar, 100 μm. **e** Immunohistochemistry analysis of centrosome amplification (γTub) and Cep131 expression. Scale bar, 10μm. Quantification of Cep131 overexpression (**f**) and centrosome amplification (**g**) in tumor tissues. Error bars represent means ± SD from four tissue sections (*N* = 2 for each group). *P < 0.05, unpaired Student’s *t* test

To further verify the role of Cep131 expression in colon cancer progression, HCT116 cells, deficient in or stably expressing Cep131 (WT and 2A), were divided into four groups according to Cep131 expression levels (Fig. S9a–c). Next, Cep131-expressing HCT116 cells were subcutaneously injected into nude mice. The xenograft assay demonstrated that Cep131-depleted cells displayed no tumor formation or growth (Fig. 7a and S9d). Similarly, tumor growth in a breast carcinogenesis model was greatly suppressed by Cep131 depletion^27^. Furthermore, Cep131 overexpression accelerated tumor growth between 3 and 4 weeks. At 5 weeks, tumor volumes and growth in the Cep131-WT expressing group did not noticeably differ from those in the control group, as the tumors reached maximum size (Fig. 7b, c). However, overexpression of Cep131-2A did not accelerate tumor growth, and even decreased tumor weight (Fig. 7b, c).

We then subjected the tumors to H&E staining and immunohistochemistry. Staining showed that tumor tissues in each group were not presented as colorectal cancer structure (Fig. 7d), as these are xenograft mouse models. However, tumor tissues revealed an increase of a homogeneous cell population, including the high proportion of the cytosol and the nucleus, in spite of different tumor sizes in each group (Fig. 7a, d), reflecting the histological feature of tumor tissue. Moreover, immunohistochemistry analysis revealed excessive accumulation of Cep131 at the centrosome in the Cep131-WT and 2A groups (Fig. 7e, f). As expected, centrosome amplification was significantly increased only in the Cep131-WT group (Fig. 7g). Thus, Cep131 phosphorylation by Plk4 is critical for centrosome amplification and for cancer progression.

## Discussion

**Fig. 8:**
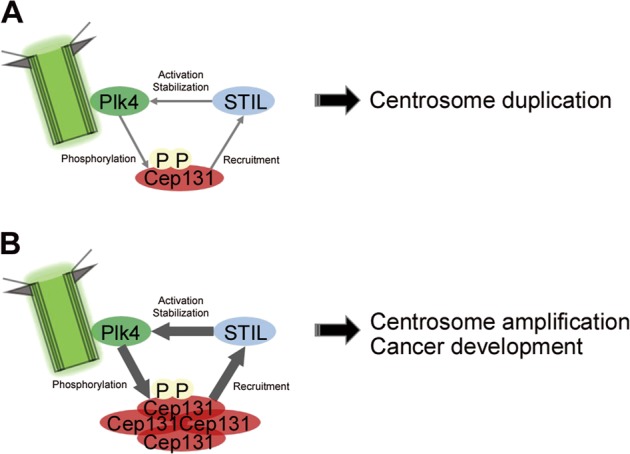
Model suggesting Cep131 as a mediator of STIL recruitment and Plk4 stability. **a** At the beginning of centrosome duplication, Plk4 phosphorylates not only STIL but also Cep131 at residues S21 and T205. Cep131 phosphorylation facilitates STIL recruitment to elongate new procentrioles. **b** Cep131 overexpression increases excessive recruitment of STIL at the centriole, which leads to uncontrolled stabilization and activation of Plk4. This coordinated cycle promotes centrosome amplification and therefore cancer progression

Plk4 stability and activity must be controlled for proper centrosome duplication^10,12,17–19^. Plk4 maintenance and activity during initiation of centrosome duplication are required for interaction with STIL, which enhances Plk4 kinase activity and protects it from proteolytic degradation^13–15,21^. We found that Plk4 directly interacts with Cep131 through specific PBM residues and phosphorylates Cep131 at residues S21 and T205, although S21 was the more suitable phosphorylation site. Our study showed that Cep131 phosphorylation facilitates interaction with STIL and recruits it to the centriole. Moreover, Cep131 overexpression caused excessive STIL recruitment, concurrently accumulating and stabilizing Plk4 and leading to centrosome amplification and cancer development. This suggests that elevated Cep131 levels induced by loss of USP9X caused centrosome amplification and promoted breast cancer progression^27^. These findings all support our suggested model of a positive feedback loop, illustrating that centrosome duplication might be initiated by activated Plk4, which phosphorylates Cep131, through which STIL is recruited to the centriole (Fig. 8).

STIL not only promotes Plk4 activation and stabilization, but is also required for Plk4 kinase activity, which localizes STIL to the centriole, implying that the proteins affect one another in a positive feedback loop^15,22,39^. In this study, we observed that the Cep131 levels affect centriolar abundance of STIL. Cep131 depletion slightly reduced STIL at the centriole, and Cep131re-expression reversed this. In contrast, mutation of the Plk4-dependent phosphorylation sites in Cep131 to alanine residues did not influence STIL localization. Thus, our observations support previous evidence that the essential role of Plk4 kinase activity is localizing STIL to the centriole by demonstrating that Cep131 phosphorylation by Plk4 facilitates recruitment of STIL by a phosphorylation-dependent Cep131/STIL interaction. Considering the role of Cep131 in centrosome duplication as well as its physical association with Plk4 and STIL, it is possible that Cep131 acts as a mediator in STIL recruitment to the centriole and Plk4 stabilization during the initiation of centrosome duplication.

Although previous studies attempted to clarify the association of centrosome amplification with cancer development, whether centrosome amplification-induced genomic instability, such as aneuploidy, directly promotes tumor progression is controversial^3,40–43^. In this study, centrosome amplification after Cep131 overexpression in HCC116 cells accelerated tumor formation and growth in nude mice, suggesting a relationship between centrosome amplification and tumor development. Furthermore, previous studies reported that modest increases in Plk4 levels induce small increases in centrosome amplification, approximately one or two centrosomes per cell, which were favorable condition for spontaneous tumorigenesis in a mouse model^7^. Strong Plk4 expression, which stimulates profound centrosome amplification, may be detrimental to cell growth due to substantial multipolar mitosis rather than overproliferation and/or tumor development^7^, and therefore might not induce spontaneous tumorigenesis^6,44,45^. Consistently, we observed that Cep131 overexpression in U2OS cells slightly elevated the Plk4 signal intensity at the centriole by ~1.5-fold, compared with that in the control group, and produced one or two extra centrosomes per cell. Moreover, Cep131 overexpression increased centrosome amplification by ~10%, much less than did Plk4 overexpression (~50%). We therefore propose that Cep131 overexpression provide favorable condition to accelerate tumor progression by moderately increasing centrosome amplification.

In conclusion, we discovered a novel function of Cep131 as a critical mediator of Plk4 stability during initiation of centrosome duplication. Although Cep131 is not essential to the centriole structure, its dysregulation poses a risk of centrosome amplification and therefore cancer development. These findings clarify the complex link between centrosome number and cancer development, and identify Cep131 as a prospective molecular target in cancer therapy and diagnosis.

## Materials and methods

### Cell culture, transfection, and stable cell lines

Human U2OS and HEK293T cells were obtained from the American Type Culture Collection (ATCC, Manassas, VA, USA) and cultured according to manufacturer recommendations in Dulbecco’s Modified Eagle’s Medium (DMEM) supplemented with 10% fetal bovine serum (Thermo Fisher Scientific, Waltham, MA, USA). Cells were transfected using either X-tremeGENE HP DNA Transfection Reagent (Roche, Basel, Switzerland) for protein expression or Oligofectamine (Invitrogen, Carlsbad, CA, USA) for siRNA transfection.

To generate lentiviruses for stable cell lines, including protein expression or knockdown, pHR’-CMV-R 8.2 delta vpr and pHR’-CMV-VSV-G (protein G of vesicular stomatitis virus) were transfected into 293T cells containing pHR’-CMV-vector, Plk4, and Cep131 for stable expression or pLKO.1 puro-shGL2 (luciferase, control), shPlk4, shSTIL, and shCEP131 for knockdown. U2OS and HCT116 cells were infected with the indicated viruses and treated with 2 μg/ml puromycin for 2 days to select a lentivirus-integrated population. The sequences used for siRNA or shRNA are listed in Table S3.

### DNA constructs

DNA construct including pcDNA-3×-Myc-Plk4 WT mutant were received from Addgene (Cambridge, MA, USA). A kinase-dead mutant (KD, K41R) and non-degradable (constitutively active form, S285A and T289A) Plk4 was generated by RT-PCR-based mutagenesis. pGEX6p-1-GST-Plk4 WT and K41R were produced by inserting BamH I-Xho I fragments into the pGEX6p-1 vector. To generate tetracycline-inducible stable cell lines, both Myc-Plk4 WT and K41R mutant were inserted into pTet-IRES-EGFP vector using BamH I-Xho I fragments.

pCI-Neo constructs encoding HA-Cep131 were purchased from Open Biosystems (Huntsville, AL, USA), and then alanine substitution mutants and phosphomimetic mutants were created by RT-PCR-based mutagenesis. The expression constructs for HA-Cep131 including deletion mutants were generated with a Q5 site-directed mutagenesis kit (NEB).

pEGFP-C1-STIL (isoform 4) was produced by RT-PCR and analyzed by sequencing. Alanine substitution mutant, including S1015, S1062, and S1070 (counterpart of S1061, S1108, and S1116 in isoform 1), were created by RT-PCR-based mutagenesis.

### Antibodies

Rabbit polyclonal antibodies against Cep131 (A301-415A, immunofluorescence (IF) 1:100, immunoblotting (IB) 1:2,000; Bethyl Laboratories, Montgomery, TX, USA), STIL (A302-441A, IF 1:100, IB 1:1,000; Bethyl Laboratories) (ab89314; immunoprecipitation (IP) 2 mg/ml; Abcam, Cambridge, UK;), Plk4 (ab1373398, IB 1:1,000, IP 2 mg/ml; Abcam), γ-tubulin (T5192, IF 1:500, IB 1:3,000; Sigma-Aldrich, St. Louis, MO, USA) HA-tag (Y-11, sc-805, IF 1:100, IB 1:2,000; Santa Cruz Biotechnology; Dallas, TX, USA), and GFP-tag (FL, sc-8334, IB 1:2,000; Santa Cruz Biotechnology), as well as mouse monoclonal antibodies against centrin (20H5, 04-1624, IF 1:100; EMD Millipore, Billerica, MA, USA), SAS-6 (91.390.21, sc-81431, IF 1:100; Santa Cruz Biotechnology), ninein (clone 79-160, MABT29, IF 1:200; EMD Millipore), γ-tubulin (T6557, IF 1:500, IB 1:3,000; Sigma-Aldrich), and c-Myc (9E10, sc-40, IB 1:2,000; Santa Cruz Biotechnology) were used and purchased as indicated.

To generate specific antibodies against Plk4 (residues 546–579) and phosphorylated Cep131 (pS21), peptides were synthesized from residues 546–579 (MTALHSKPEIIQQECVFGSDPLSEQSKTRGMEPP) and from residue 16–25 (AGVDLpSLTGL), respectively. All antibody production, including immunization, serum collection, and antibody purification, was carried out by Younginfrontier (Seoul, Korea). The rabbit Plk4 antibody was used at 1:100 dilutions for IF, and the Cep131-pS21 antibody was used at 1:100 dilutions for IF or 1:1,000 dilutions for IB.

### Immunofluorescence microscopy and immunoblotting

U2OS cells were grown on poly-l-lysine (Sigma-Aldrich)-coated cover glasses. For signal extraction, cells were incubated with extraction buffer (80 mM PIPES, pH 6.8, 1 mM EGTA, 1 mM MgCl_2_, and 10% NP-40) for 30 s, and then fixed with −20 °C methanol for 10 min. After blocking in PBS with 5% BSA for 1 h, cells were incubated with primary antibodies for 2 h, washed with PBS three times, and incubated with Alexa Fluor 488 or 633 or Texas red-conjugated secondary antibodies (Invitrogen). To visualize DNA, cells were stained with 0.1 µg/ml of Heochst33342 (Sigma-Aldrich), and then mounted onto slide glasses. Fluorescence images were collected with a Carl Zeiss LSM 710 confocal microscope or with a Carl Zeiss Axiovert 100M microscope (Carl Zeiss AG, Oberkochen, Germany). For super-resolution imaging, an Olympus IX83 (100×; Olympus, Shinjuku, Japan) with detector (iXon 897 Life with SRRF-Stream; Andor, Belfast, Ireland) was used. Counting of cells with fluorescence signals and quantification of signal intensities were performed using the ZEN lite software (Carl Zeiss).

For immunoblot analyses, protein samples were separated by SDS-PAGE, transferred to PVDF membranes (Bio-Rad, Hercules, CA, USA), and blocked with 5% skimmed milk in TBS containing 0.05% Tween (Sigma). The membranes were then incubated with primary antibodies as described above, followed by incubation with rabbit- or mouse-horseradish peroxidase-linked secondary antibodies (Cell Signaling Technology, Danvers, MA, USA). Proteins were detected using an Enhanced Chemiluminescence detection system (Thermo Fisher Scientific).

### Immunoprecipitation

Protein samples were extracted from U2OS or HEK293T cells by incubation in TBSN buffer (50 mM Tris–HCl, pH 8.0, 120 mM NaCl, 5 mM EGTA, 1.5 mM EDTA, and 0.5% NP-40) with a protease inhibitor cocktail (Roche) for 20 min on ice. Whole lysates were incubated with anti-GFP- or HA- or Myc-tagged magnetic beads (Thermo Fisher Scientific) for 2 h at 4 °C. After binding, the magnetic beads were washed with TBSN five times, and the proteins were separated by SDS-PAGE.

### Mass spectrometry analyses

To identify Cep131 phosphorylation, fragments of phosphorylated Cep131 were separated by SDS-PAGE, and the gel bands were excised. Reduction with dithiothreitol (DTT, Sigma-Aldrich) and alkylation with indole-3 acetic acid (IAA, Sigma-Aldrich) were performed before each gel band was treated with trypsin (Promega, Madison, WI, USA) to digest the proteins in situ^46^. Bands were washed with 10 mM ammonium bicarbonate and 50% acetonitrile (ACN; Honeywell, Wabash, IN, USA); swollen in digestion buffer containing 50 mM ammonium bicarbonate, 5 mM CaCl_2_, and 1 µg of trypsin; and then incubated at 37 °C for 16 h. Peptides were recovered by two cycles of extraction with 50 mM ammonium bicarbonate and 100% ACN. The resulting peptide extracts for each band were lyophilized and stored at −20 °C until MS analysis.

Peptides were analyzed using one-dimensional liquid chromatography/tandem mass spectrometry (1DLC–MS/MS). Peptides were identified using MS/MS with a nano-LC–MS system consisting of a nanoACQUITY UltraPerformance LC System (Waters, Milford, MA, USA) and an LTQ Orbitrap Elite mass spectrometer (Thermo Fisher Scientific) equipped with a nano-electrospray source.

An autosampler was used to load 5-µl aliquots of the peptide solutions onto a C18 trap column (id 180 μm, length 20 mm, and particle size 5 μm; Waters). The peptides were desalted and concentrated on the column at a flow rate of 4 µl/min for 10 min. Then, the trapped peptides were back-flushed and separated on a 150-mm homemade microcapillary column consisting of C18 (Aqua, particle size 3 µm) packed into 100-µm silica tubing with an orifice i.d. of 5 µm. The mobile phases A and B were composed of 0 and 80% acetonitrile, respectively, and each contained 0.02% formic acid and 0.5% acetic acid. The LC gradient began with 5% B for 15 min, and was ramped to 15% B over 5 min, to 50% B over 70 min, and to 95% B over 5 min, and remained at 95% B over 5 min and 5% B for another 5 min. The column was re-equilibrated with 5% B for 15 min before the next run. The voltage applied to produce an electrospray was 2.2 kV. In each duty cycle of mass analysis, one high-mass resolution (100,000) spectrum was acquired using the FT-ICR analyzer, followed by five data-dependent MS/MS scans using the linear ion trap analyzer. For MS/MS analysis, normalized collision energy (35%) was used throughout the collision-induced dissociation phase. Previously fragmented ions were excluded for 60 s.

MS/MS spectra were analyzed using the following software analysis protocols with the Uniprot human database. The reversed sequences of all proteins were appended into the database for calculation of false discovery rate. ProLucid^47^ was used to identify the peptides, a precursor mass error of 25 ppm, and a fragment ion mass error of 600 ppm. Trypsin was selected as the enzyme, with one potential missed cleavage. Carbamidomethylation at cysteine was chosen as a static modification. Oxidation at methionine and phosphorylation at serine, threonine, and tyrosine were chosen as variable modifications. The output data files were filtered and sorted to compose the protein list using the DTASelect (Scripps Research Institute, San Diego, CA, USA)^48^ with two or more peptide assignments for protein identification and a false-positive rate of <0.01. Finally, all phosphopeptides identified in MS/MS spectral data were manually analyzed for peptide identification.

Nano-LC–MS/MS analysis was performed at the Korea Basic Science Institute (Ochang, Division of Biomedical Omics Research).

### Tumor xenograft assay

HCT116 cells were infected and divided into four groups: Vector with shGL (control), Vector with shCep131, Cep131-WT with shCep131, and Cep131-2A with shCep131. After verifying Cep131 expression, 1 × 10^6^ cells in 150 μl of PBS were subcutaneously injected into 7-week-old nude mice (BABL/c nude, Orient Bio, Seongnam, Korea). Tumor tissues were measured every week, and volume was calculated according to the formula π/6 × length × width^2^. After 5 weeks, the mice were killed, and tumors were collected for further study.

### Histology

Tumor tissues were fixed in 4% PFA overnight at 4 °C. After paraffinization, samples for hematoxylin–eosin staining and immunohistochemistry were cut by a microtome into 4–6-μm sections and plated onto slide glasses.

For immunohistochemistry, paraffin sections were deparaffinized and placed in Antigen Unmasking Solution (citric base; Vector Laboratories, Burlingame, CA, USA) for 20 min with boiling. After being rinsed three times with PBS, the sections were incubated with 5% BSA for blocking. They were then incubated with primary antibodies, such as anti-γ-Tub and Cep131 overnight at 4 °C. After being rinsed with PBS three times, the sections were incubated with Alexa488 and Texas red-conjugated secondary antibodies. DNA was visualized by staining with 0.1 µg/ml of Heochst33342.

### Gene database

A functional study of the gene interaction network was performed using Ingenuity Pathway Analysis (Ingenuity Systems). Genome-wide expression data for cancer samples and cell lines were acquired and analyzed on the Oncomine website (https://www.oncomine.org) to search the Cep131 gene expression level with significant statistical values (*P*-value < 0.001 and fold change 1.5). Cep131 protein expression data in several organs were obtained from the Human Protein Atlas (https://www.proteinatlas.org). To analyze expression of the gene in more detail, expression profiling from TCGA RNAseqV2 data and RSEM^49^ values (https://deweylab.github.io/RSEM/) were used. After collecting tumors and the matched normal data set for each cancer type, mRNA expression levels were compared and matched to normal isoforms.

## Supplementary information

Supplemental Figure legends and Tables

Supplemental Figures (1-9)
