# Supplementary material for: Cep131 overexpression promotes centrosome amplification and colon cancer progression by regulating Plk4 stability
Source: Cell Death Dis. 2019 Jul 29;10(8):570. doi: 10.1038/s41419-019-1778-8 (PMC6662699; doi:10.1038/s41419-019-1778-8)
Supplement: Supplementary file 1 — Supplemental Figure legends and Tables [file 41419_2019_1778_MOESM1_ESM.pdf]

Supplemental Information

Cep131 Overexpression Promotes Centrosome Amplification and Colon Cancer Progression by Regulating Plk4 Stability

Kim et al.

Supplementary Figure legends

Figure S1 Cep131-induced centrosome amplification is closely associated with Plk4. a Immunofluorescence analysis of the localization of exogenous Cep131 at the centrosome. U2OS cells were stained with anti-Cep131 or HA (red) antibodies. Scale bar, 10 μm. **b** Immunoblotting and RT-PCR analysis show the protein and RNA levels of Plk4, respectively. U2OS cells were transfected with siCon or siPlk4 duplex. **c** Quantification of centrosome amplification in HeLa and U2OS cells. Error bars represent means ± SEM from three independent experiments (N>300 for each experiment). **P<0.01, unpaired Student's t-test. **d** Immunoblotting analysis shows protein levels of Cep131 in U2OS cells transfected with siCon or siCep131 duplexes. **e** Immunoblotting analysis shows doxycycline-inducible expression of Myc-Plk4 WT and KD (kinase dead) in U2OS cells. **f** Quantification of centriole and centrosome amplification. U2OS cells with doxycycline-inducible expression of Myc-Plk4 WT and KD were transfected with siCon or siCep131. Error bars represent means ± SEM from three independent experiments (N>300 for each experiment). *P<0.05 and **P<0.01, unpaired Student's t-test. CBB staining use as a loading control in **b**, **d**, and **e**.

Figure S2 Cell cycle-dependent localization of Cep131 at the centriole. a and b Immunofluorescence analysis of U2OS cells at different phases in the cell cycle using anti-Cep131 co-stained with γTub **a** or Cent **b**. The cell cycle phases were distinguished by DNA (blue). Insets are approximately 5-fold magnified at the centrosomal region. Scale bar, 10 μm. **c** Immunofluorescence analysis of direct localization of exogenous Cep131 at the centriole. U2OS cells expressing GFP-Cep131 (green) were stained with Cent or Plk4. Scale bar, 10 μm. **d** Immunofluorescence analysis of co-localization of Plk4 with Cent at the centriole. U2OS cells were transfected with siCon or siPlk4, and were stained with anti-Plk4 and Cent (top panel) or Cep131 (bottom panel). Scale bar, 10 μm.

Figure S3 Asymmetrical localization of Cep131 at centrioles. a Immunofluorescence analysis of

U2OS cells with asymmetrical localization of Cep131 at centrioles. Cep131 co-stained with Cent (far-red) and mother centriole-enriched protein, Ninein (green). Schematic illustration of the asymmetrical localization of Cep131 at each centriole. M, mother centriole; D, daughter centriole. Scale bar, 0.5 μm. **b and c** U2OS cell transfected with siRNAs against Cep152, Cep192, ATF5, hCenexin, Cep250, Rootletin, Plk4, and STIL, were then fixed and stained with Cent and Cep131. Quantification of fluorescence intensity of Cep131 at the centrioles **b**. Over 35 centrioles from three independent experiments were measured for each condition. Scale bar, 10 μm.

Figure S4 Physical interaction between Cep131 and Plk4. a Schematic representation of the protocol for discovering Plk4 binding sites in Cep131. The coiled coil domains in Cep131, indicated in blue, were analyzed by using the UniProtKB/Swiss-Prot database (http://www.uniprot.org/align/). **b-d** Immunoblotting analysis of immunoprecipitated samples from HEK293T cells co-expressing Myc-Plk4 with several fragments of Cep131, as shown in **a**. The fragments of Cep131, which interact with Plk4, are indicated in red. **e** Schematic representation of deletion mutants in Cep131 residues 513–542. **f** HEK293T cells co-expressing Myc-Plk4 and HA-Cep131 fragments, as shown in **e**, were immunoprecipitated. The proteins were analyzed by immunoblotting. **g** Schematic representation of Cep131-binding domain in Plk4. Fragments of Plk4 were generated depending on the Plk4 domain, including kinase domain, PB1, PB2, and PB3. **h** HEK293T cells were transfected with HA-Cep131 and Myc-Plk4 fragments, and then immunoprecipitated using HA-magnetic beads. CBB staining use as a loading control in **b**, **c**, **d**, **f**, and **h**.

Figure S5 Cep131 is phosphorylated by Plk4 at residues S21 and T205. a Bacterially expressed His-Cep131 fragments, such as N (1-384), M (363-739), and C (711-1081) were incubated with GST-Plk4-WT or KD, which was also purified from bacteria, for 30 min at 30 °C. The incorporation of γ-P32 ATP as substrates was visualized by autoradiography. **b** MS/MS spectra of phosphorylated Cep131 at residues S21 and T205. **c** In vitro kinase assay with GST-Plk4 and alanine mutants of mHis-Cep131-N,

including S21A, T23A, T205A, and S21+T205A (2A), which underwent Plk4-dependent phosphorylation, as determined by MS analysis. **d** Alignment of amino acids in human Cep131 and several other species. The phosphorylation sites at residues T205 by Plk4 are highlighted in red. **e** HEK293T cells co-expressing Myc-Plk4-ND and HA-Cep131 WT, KD, or ΔPBM were immunoprecipitated using HA-magnetic beads. Phosphorylation status was observed by immunoblotting using anti-Cep131-pS21 antibody. CBB staining use as a loading control in **a**, **c**, and **e**.

Figure S6 Phosphorylated Cep131 interacts with STIL. **a** HEK293T cells co-expressing GFP-STIL and the phospho-mimetic form of HA-Cep131 (S21 and T205 to D or E) were immunoprecipitated using GFP-magnetic beads. CBB staining use as a loading control. **b** Immunofluorescence analysis of direct localization of Cep131 alanine mutants at the centriole. U2OS cells stably expressing untagged Cep131 WT, S21A, T23A, T205A, and 2A were transfected with siCep131 to remove endogenous Cep131, and then stained with anti-Cent (green) and anti-Cep131 (red) antibodies.

Figure S7 Cep131 overexpression leads to stabilization of Plk4. **a** HEK293T cells co-expressing Myc-Plk4 with HA-Ub and GFP-Cep131 or GFP-STIL were immunoprecipitated. Ubiquitylation properties were analyzed by immunoblotting. **b** Endogenous Plk4 extracted from U2OS cells transiently expressing HA-Cep131 WT or 2A was immunoprecipitated. MG132, a protease inhibitor, were administered for 6 h. **c** U2OS cells stably expressing untagged Cep131 WT or 2A, which contained a resistant sequence to siRNA, were transfected with siCon or siCep131 to remove endogenous Cep131. **d** Quantification of centriole and centrosome amplification in U2OS cells with expression as indicated in **c**. Error bars represent means ± SEM from three independent experiments (N>300 for each experiment). **P<0.01, unpaired Student's t-test. CBB staining use as a loading control in **a-c**.

Figure S8 Cep131 Gene expression in cancer. a A disease summary for Cep131 in normal and cancer samples and mRNA expression with P-value <0.001 using the Oncomine database. Notably, Cep131 is up-regulated in bladder cancer and colorectal cancer (highlighted in red). **b** The isoform expression levels of Cep131 in colon cancer (COAD; colon adenocarcinoma) on the basis of TCGA RNASeq dataset as shown in Table S2. ****P<0.0001 (according to TCGA database).

Figure S9 Cep131 overexpression promotes centrosome amplification and cancer progression. a Schematic representation of the experimental schedule used for the xenograft assay. **b** Immunoblotting analysis shows the protein levels of Cep131. HCT116 cells stably expressing untagged Cep131 WT or 2A were transfected with shGL2 (control) or shCep131 to eliminate endogenous Cep131. CBB staining use as a loading control. **c** Quantification of centriole and centrosome amplification in HCT116 cells. Error bars represent means ± SEM from three independent experiments (N>300 for each experiment). *P<0.05 and **P<0.01, unpaired Student's t-test. **d** HCT116 cells were subcutaneously transplanted. After 5 weeks, tumors had grown to maximum sizes and mice were sacrificed.

Table S1 Differential analysis of Cep131 expression in several cancer types (Oncomine, Human Genome U133 Plus 2.0 Array).

Cancer type		Fold change (Cancer vs. Normal)	P-value
Colon cancer	Rectal adenocarcinoma	1.863	4.63^{-6}
	Colon mucinous adenocarcinoma	1.93	1.42^{-7}
	Rectosigmoid adenocarcinoma	1.954	1.26^{-5}
	Cecum adenocarcinoma	1.668	4.94^{-6}
Dyrskjot bladder 3	Superficial bladder	1.838	1.06^{-9}
	Infiltrating bladder urothelial carcinoma	1.702	2.76^{-6}
Lee bladder	Superficial bladder cancer	1.539	4.79^{-7}

Table S2 Cep131 expression level in each cancer type based on average normalized read count (TCGA database)

Cancer	Average normalized read count	No. of samples
Dlbc	1016.721861	28
Ucec	841.821409	1732
Blca	673.865142	1720
Skcm	636.500778	3106
Cesc	607.27694	842
Sarc	574.598698	272
Luad	380.337825	4841
Lusc	374.922449	3040
Lgg	374.318719	1981
Hnsc	366.60644	1987
Kirp	352.399915	1206
Brca	352.225756	8129
Coad	344.416701	1904
Read	338.834924	516
Acc	316.698401	79
Thca	307.891415	4592
Kich	295.159941	181
Lihc	289.318045	1086
Gbm	279.82268	338
Laml	271.264046	296
Prad	255.979585	1738
Paad	219.586888	265
Kirc	214.450005	2204

*Abbreviations: Dlbc (diffuse large B-cell lymphoma), Ucec (uterine corpus endometrial carcinoma), Blca (bladder carcinoma), Skcm (skin cutaneous melanoma), Cesc (cervical squamous cell carcinoma), Sarc (sarcomatoid cancer), Luad (lung adenocarcinoma), Lusc (lung squamous cell carcinoma), Lgg (lower-grade glioma), Hnsc (neck squamous cell carcinoma), Kirp (kidney renal papillary cell carcinoma), Brca (breast Invasive carcinoma), Coad (colon adenocarcinoma), Read (rectum adenocarcinoma), Acc (adenoid cystic carcinoma), Thca (thyroid cancer), Kich (kidney chromophobe), Lihc (liver hepatocellular carcinoma), Gbm (glioblastoma), Laml (acute myeloid leukemia), Prad (prostate adenocarcinoma), Paad (pancreatic adenocarcinoma), and Kirc (kidney renal clear cell carcinoma).

Table S3 List of siRNA and shRNA sequences.

Targets		Sequences
siCep131	710	5′-CCCACTCAGCCCGGAACAATA-3′
	2005	5′-CAGCACGAGCTGGAGATTA-3′
siPLK4	997	5′-CAGTATAAGTGGTAGTTTA-3′
	3158	5′-AGAACAAAGCAGAATGAAA-3′
siSTIL	1114	5′-GAGAAAAGGTGTAAGCATT-3′
	2440	5′-TGTTGAACCTCCTGACAAA-3′
siPCNT	1580	5′-GTTCAAAGAGAGCGAGAAA-3′
	1847	5′-GTTAAGGGATGCTGAGAAA-3′
siRootle	3292	5′-TCAGAGAAGTTGATGGGTA-3′
	3746	5′-TGAAGAAGGCAGAGAGCGA-3′
siCep250	4221	5′-GAGCAGAGCTACAGCGAAT-3′
	3369	5′-AGAAGGAGCTGGAGAGAGA-3′
siATF5	417	5′-GCTCGTAGACTATGGGAAA-3′
	1696	5′-GGATAGAGCTGAAGGACTA-3′
sihCenexin	781	5′-AGACUAAUGGAGCAACAAG-3′
	2066	5′-GGCAGUUGGAGAGUGCCAU-3′
