# Supplementary material for: Cep131 overexpression promotes centrosome amplification and colon cancer progression by regulating Plk4 stability
Source: Cell Death Dis. 2019 Jul 29;10(8):570. doi: 10.1038/s41419-019-1778-8 (PMC6662699; doi:10.1038/s41419-019-1778-8)
Supplement: Supplementary file 2 — Supplemental Figures (1-9) [file 41419_2019_1778_MOESM2_ESM.pdf]

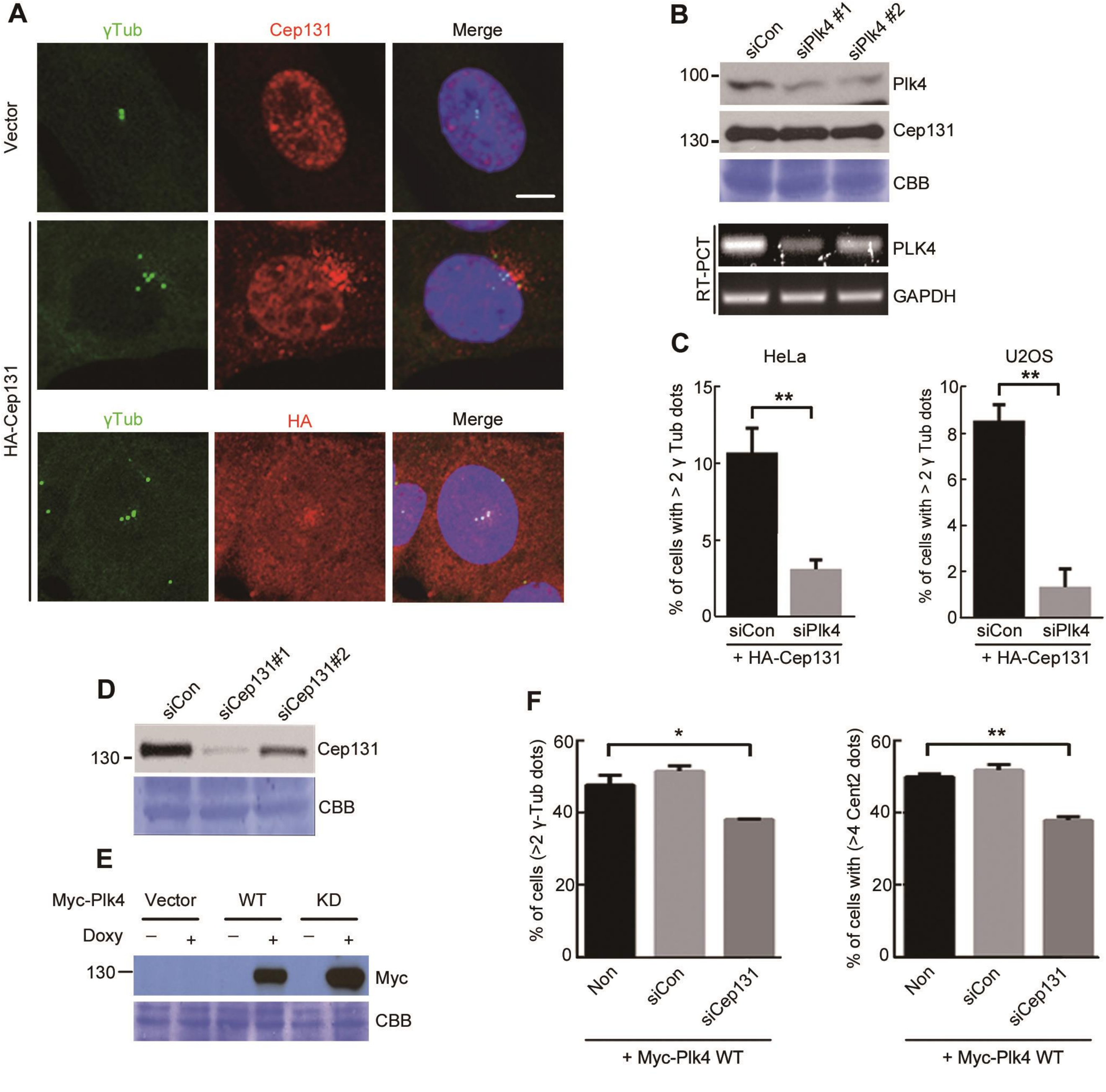
A
γTub
Cep131
Merge
Vector
HA-Cep131
γTub
HA
Merge
B
siCon
siPlk4 #1
siPlk4 #2
100
Plk4
130
Cep131
CBB
RT-PCT
PLK4
GAPDH
C
HeLa
% of cells with > 2 γ Tub dots
15
10
5
0
**
siCon
siPlk4
+ HA-Cep131
U2OS
% of cells with > 2 γ Tub dots
10
8
6
4
2
0
**
siCon
siPlk4
+ HA-Cep131
D
siCon
siCep131#1
siCep131#2
130
Cep131
CBB
E
Myc-Plk4
Vector
WT
KD
Doxy
−
+
−
+
−
+
130
Myc
CBB
F
% of cells (>2 γ-Tub dots)
60
40
20
0
*
Non
siCon
siCep131
+ Myc-Plk4 WT
% of cells with (>4 Cent2 dots)
60
40
20
0
**
Non
siCon
siCep131
+ Myc-Plk4 WT

Figure S1

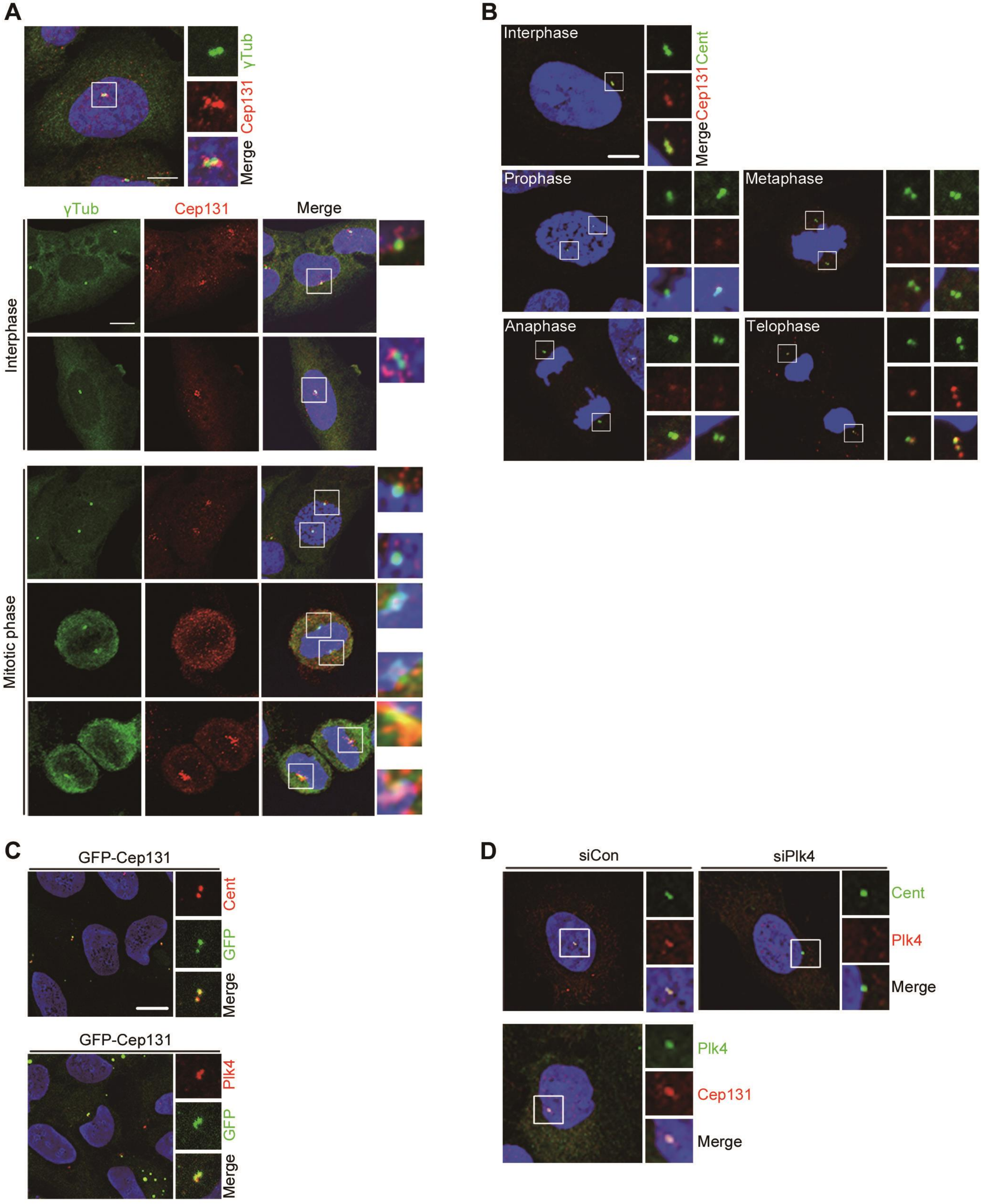
A
γTub
Cep131
Merge
γTub
Cep131
Merge
Interphase
Mitotic phase
B
Interphase
Cent
Cep131
Merge
Prophase
Metaphase
Anaphase
Telophase
C
GFP-Cep131
Cent
GFP
Merge
GFP-Cep131
Plk4
GFP
Merge
D
siCon
siPlk4
Cent
Plk4
Merge
Plk4
Cep131
Merge

Figure S2

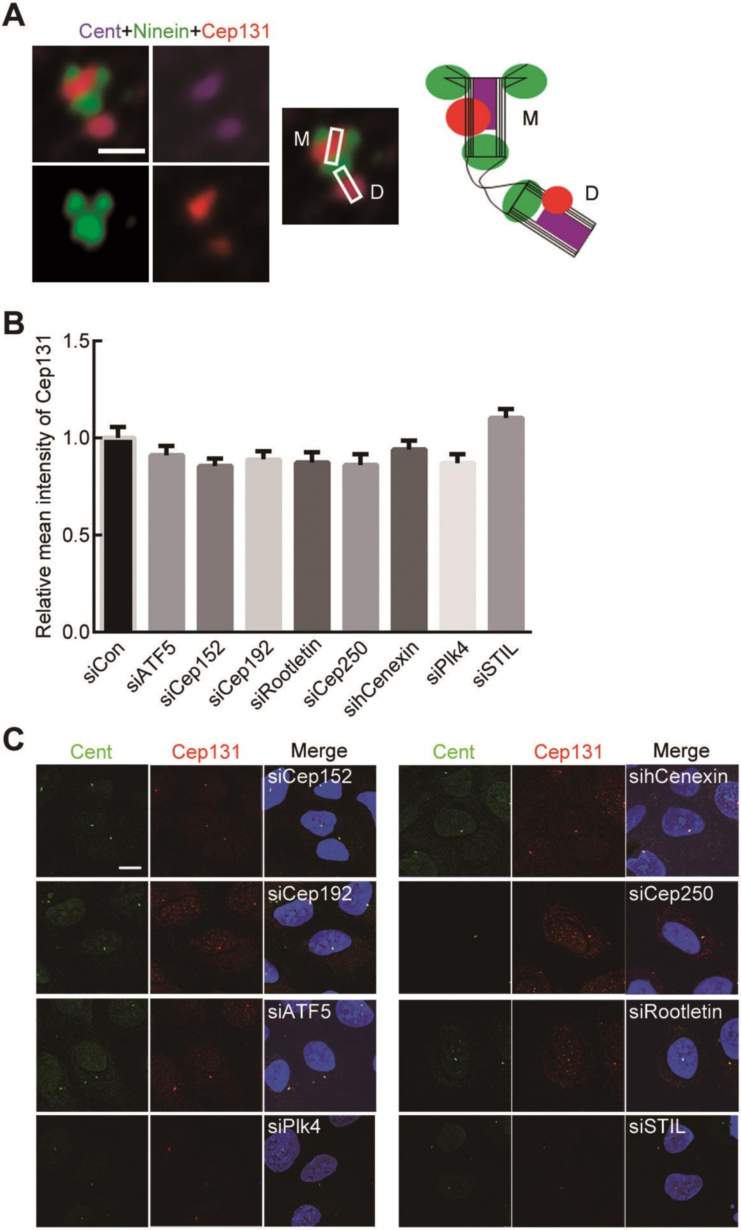
A
Cent+Ninein+Cep131
M
D
M
D
B
Relative mean intensity of Cep131
1.5
1.0
0.5
0.0
siCon
siATF5
siCep152
siCep192
siRootletin
siCep250
sihCenexin
siPlk4
siSTIL
C
Cent
Cep131
Merge
siCep152
siCep192
siATF5
siPlk4
Cent
Cep131
Merge
sihCenexin
siCep250
siRootletin
siSTIL

Figure S3

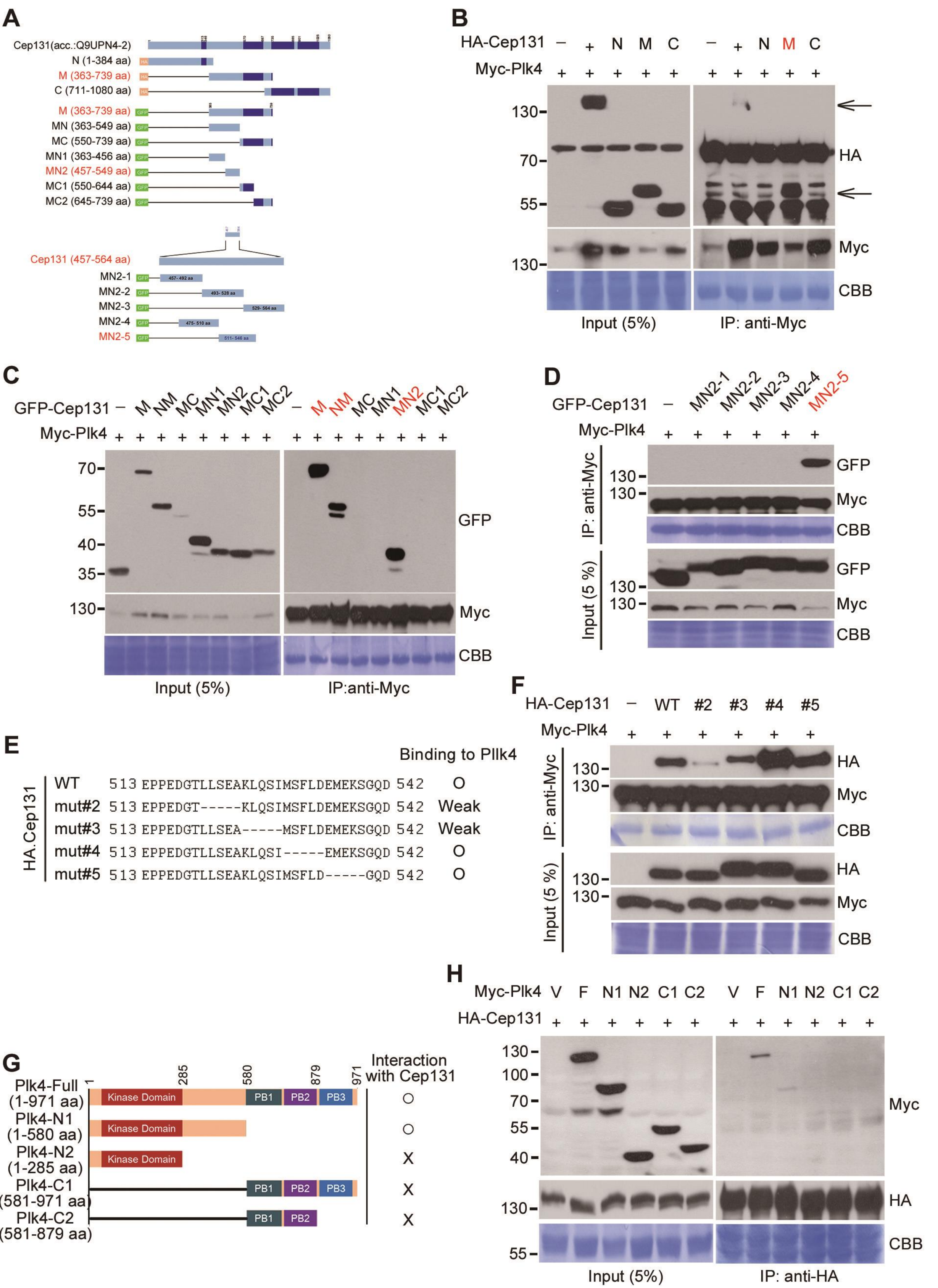
A
Cep131(acc.:Q9UPN4-2)
N (1-384 aa)
M (363-739 aa)
C (711-1080 aa)
M (363-739 aa)
MN (363-549 aa)
MC (550-739 aa)
MN1 (363-456 aa)
MN2 (457-549 aa)
MC1 (550-644 aa)
MC2 (645-739 aa)
Cep131 (457-564 aa)
MN2-1
457- 492 aa
MN2-2
493- 528 aa
MN2-3
529- 564 aa
MN2-4
475- 510 aa
MN2-5
511- 546 aa
B
HA-Cep131 – + N M C – + N M C
Myc-Plk4 + + + + + + + + + +
130
70
55
130
HA
Myc
CBB
Input (5%)
IP: anti-Myc
C
GFP-Cep131 – M NM MC MN1 MN2 MC1 MC2 – M NM MC MN1 MN2 MC1 MC2
Myc-Plk4 + + + + + + + + + + + + + + + +
70
55
40
35
130
GFP
Myc
CBB
Input (5%)
IP:anti-Myc
D
GFP-Cep131 – MN2-1 MN2-2 MN2-3 MN2-4 MN2-5
Myc-Plk4 + + + + + +
IP: anti-Myc
Input (5 %)
130
GFP
Myc
CBB
E
Binding to Pllk4
HA.Cep131
WT 513 EPPEDGTLLSEAKLQSIMSFLDEMEKSGQD 542 O
mut#2 513 EPPEDGT-----KLQSIMSFLDEMEKSGQD 542 Weak
mut#3 513 EPPEDGTLLSEA-----MSFLDEMEKSGQD 542 Weak
mut#4 513 EPPEDGTLLSEAKLQSI-----EMEKSGQD 542 O
mut#5 513 EPPEDGTLLSEAKLQSIMSFLD-----GQD 542 O
F
HA-Cep131 – WT #2 #3 #4 #5
Myc-Plk4 + + + + + +
IP: anti-Myc
Input (5 %)
130
HA
Myc
CBB
G
Interaction with Cep131
1
285
580
879
971
Plk4-Full (1-971 aa) Kinase Domain PB1 PB2 PB3 O
Plk4-N1 (1-580 aa) Kinase Domain O
Plk4-N2 (1-285 aa) Kinase Domain X
Plk4-C1 (581-971 aa) PB1 PB2 PB3 X
Plk4-C2 (581-879 aa) PB1 PB2 X
H
Myc-Plk4 V F N1 N2 C1 C2 V F N1 N2 C1 C2
HA-Cep131 + + + + + + + + + + + +
130
100
70
55
40
130
55
Myc
HA
CBB
Input (5%)
IP: anti-HA

Figure S4

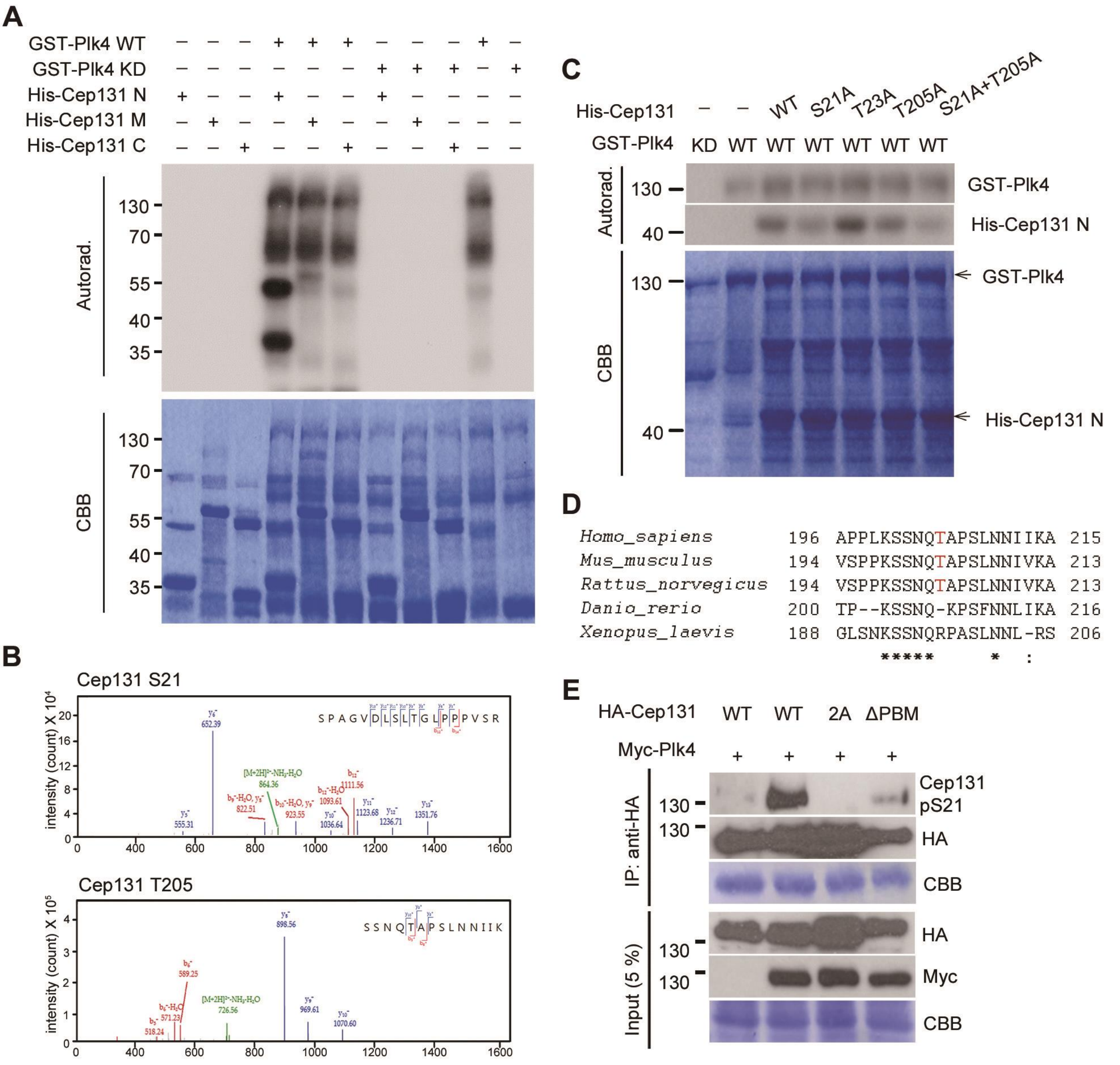
A
GST-Plk4 WT
GST-Plk4 KD
His-Cep131 N
His-Cep131 M
His-Cep131 C
Autorad.
CBB
130
70
55
40
35
B
Cep131 S21
SPAGVDLSLTGLPPPVSR
intensity (count) X 10^4
Cep131 T205
SSNQTAPSLNNIIK
intensity (count) X 10^5
0 400 600 800 1000 1200 1400 1600
C
His-Cep131 – – WT S21A T23A T205A S21A+T205A
GST-Plk4 KD WT WT WT WT WT WT
Autorad.
CBB
GST-Plk4
His-Cep131 N
D
Homo_sapiens 196 APPLKSSNQTAPSLNNIIKA 215
Mus_musculus 194 VSPPKSSNQTAPSLNNIVKA 213
Rattus_norvegicus 194 VSPPKSSNQTAPSLNNIVKA 213
Danio_rerio 200 TP--KSSNQ-KPSFNNLIKA 216
Xenopus_laevis 188 GLSNKSSNQRPASLNNL-RS 206
***** * :
E
HA-Cep131 WT WT 2A ΔPBM
Myc-Plk4 + + + +
IP: anti-HA
Input (5 %)
Cep131 pS21
HA
CBB
Myc

Figure S5

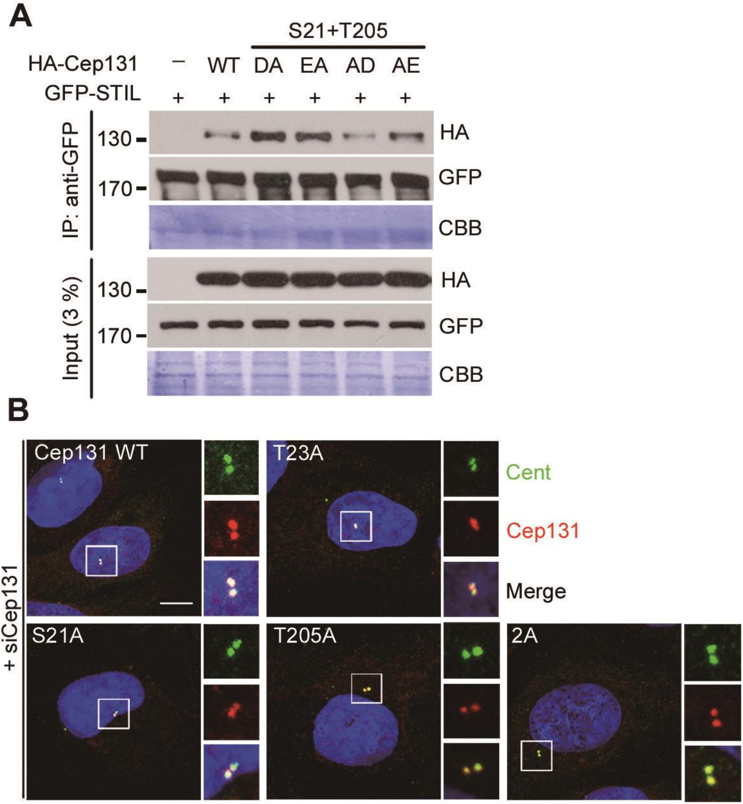
A
S21+T205
HA-Cep131
—
WT
DA
EA
AD
AE
GFP-STIL
+
+
+
+
+
+
IP: anti-GFP
130
170
HA
GFP
CBB
Input (3 %)
130
170
HA
GFP
CBB
B
+ siCep131
Cep131 WT
T23A
S21A
T205A
2A
Cent
Cep131
Merge

Figure S6

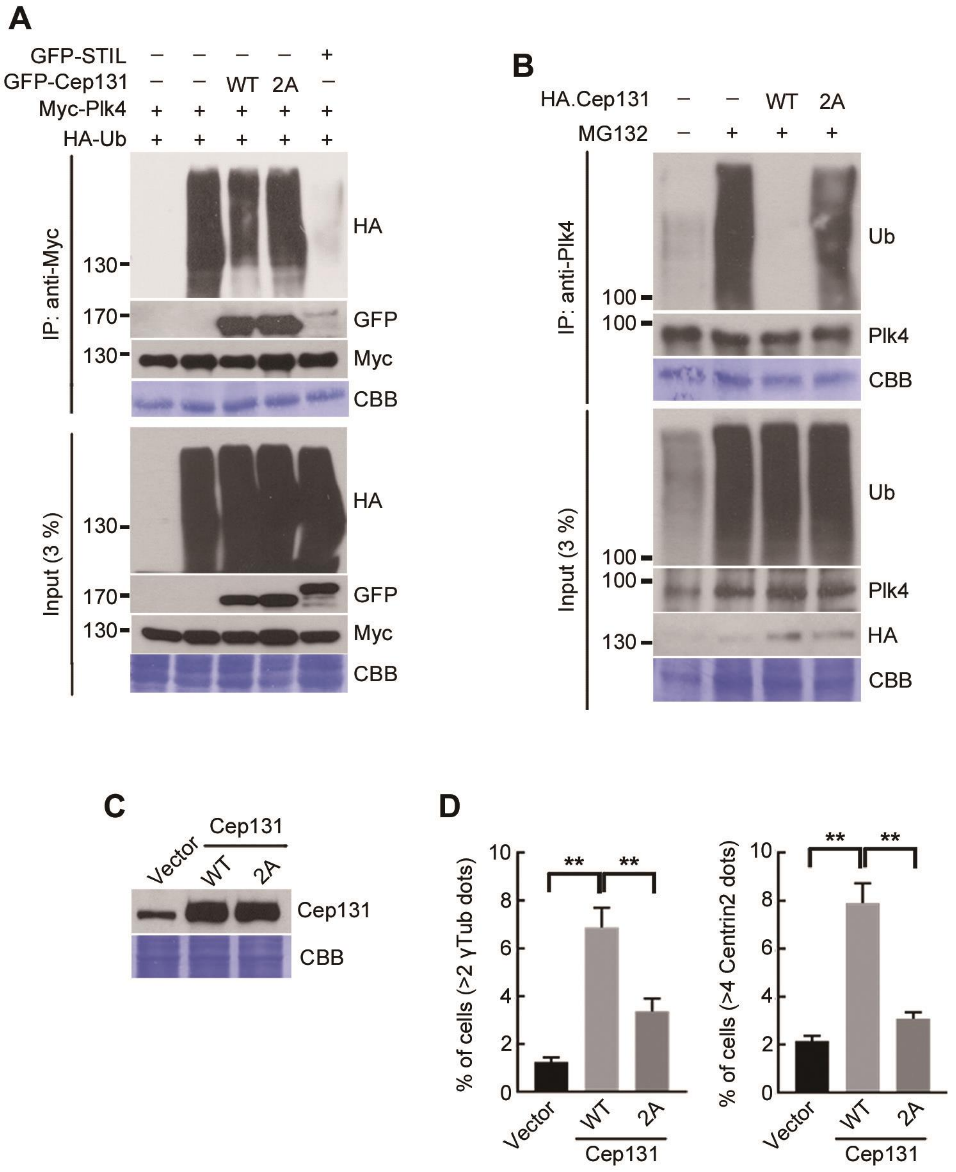
A
GFP-STIL – – – – +
GFP-Cep131 – – WT 2A –
Myc-Plk4 + + + + +
HA-Ub + + + + +
IP: anti-Myc
130
HA
170
GFP
130
Myc
CBB
Input (3 %)
130
HA
170
GFP
130
Myc
CBB
B
HA.Cep131 – – WT 2A
MG132 – + + +
IP: anti-Plk4
100
Ub
100
Plk4
CBB
Input (3 %)
100
Ub
100
Plk4
130
HA
CBB
C
Cep131
Vector
WT
2A
Cep131
CBB
D
% of cells (>2 γTub dots)
% of cells (>4 Centrin2 dots)
0
2
4
6
8
10
**
**
Vector
WT
2A
Cep131

Figure S7

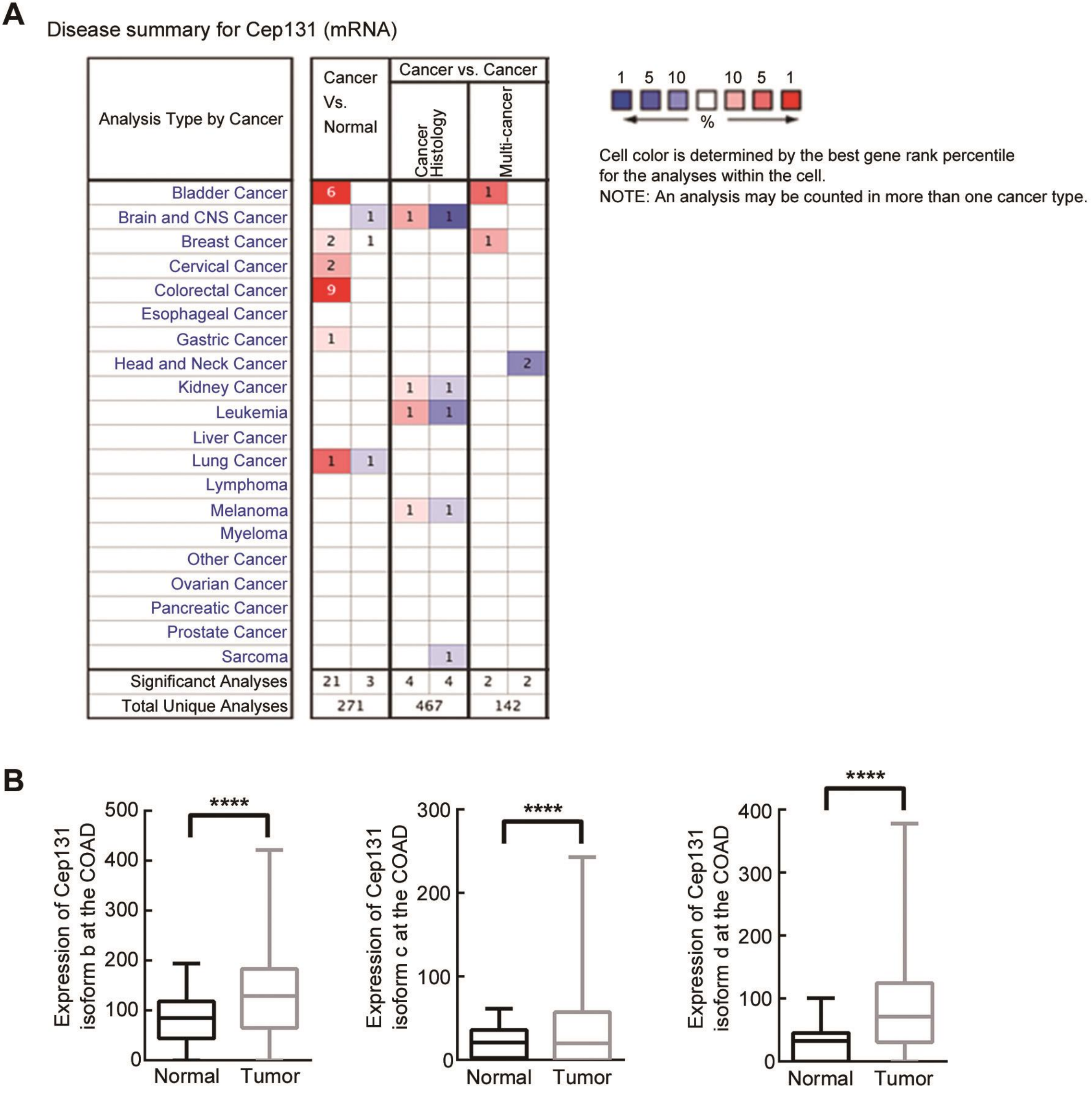
A
Disease summary for Cep131 (mRNA)
Analysis Type by Cancer
Cancer Vs. Normal
Cancer vs. Cancer
Cancer Histology
Multi-cancer
Bladder Cancer
Brain and CNS Cancer
Breast Cancer
Cervical Cancer
Colorectal Cancer
Esophageal Cancer
Gastric Cancer
Head and Neck Cancer
Kidney Cancer
Leukemia
Liver Cancer
Lung Cancer
Lymphoma
Melanoma
Myeloma
Other Cancer
Ovarian Cancer
Pancreatic Cancer
Prostate Cancer
Sarcoma
Significanct Analyses
Total Unique Analyses
21 3 4 4 2 2
271 467 142
1 5 10 10 5 1
%
Cell color is determined by the best gene rank percentile
for the analyses within the cell.
NOTE: An analysis may be counted in more than one cancer type.
B
Expression of Cep131 isoform b at the COAD
Expression of Cep131 isoform c at the COAD
Expression of Cep131 isoform d at the COAD
****
Normal
Tumor

Figure S8

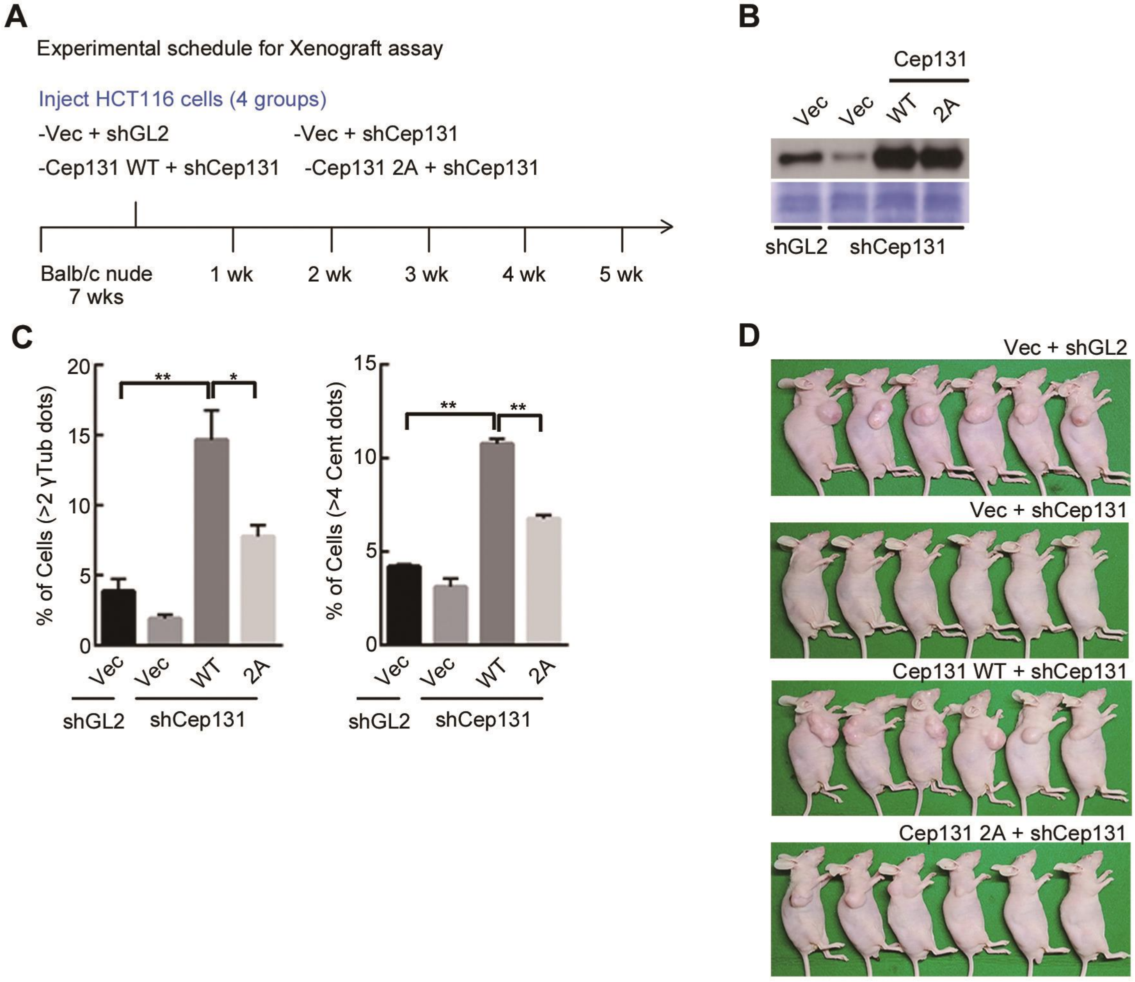
A
Experimental schedule for Xenograft assay
Inject HCT116 cells (4 groups)
-Vec + shGL2
-Vec + shCep131
-Cep131 WT + shCep131
-Cep131 2A + shCep131
Balb/c nude 7 wks
1 wk
2 wk
3 wk
4 wk
5 wk
B
Cep131
Vec
Vec
WT
2A
shGL2
shCep131
C
% of Cells (>2 γTub dots)
20
15
10
5
0
**
*
Vec
Vec
WT
2A
shGL2
shCep131
% of Cells (>4 Cent dots)
15
10
5
0
**
**
Vec
Vec
WT
2A
shGL2
shCep131
D
Vec + shGL2
Vec + shCep131
Cep131 WT + shCep131
Cep131 2A + shCep131

Figure S9
